# Non-classical monocytes promote neurovascular repair in cerebral small vessel disease associated with microinfarctions via CX3CR1

**DOI:** 10.1177/0271678X231183742

**Published:** 2023-06-21

**Authors:** Sarah Lecordier, Romain Menet, Anne-Sophie Allain, Ayman ElAli

**Affiliations:** 1 Neuroscience Axis, Research Center of CHU de Quebec – Université Laval, Quebec City, QC, Canada; 2 Department of Psychiatry and Neuroscience, 4440 Faculty of Medicine, Université Laval, Quebec City, QC, Canada

**Keywords:** Cerebral small vessel disease (cSVD), monocytes, neuroinflammation, neurovascular functions, vascular dementia (VaD)

## Abstract

Cerebral small vessel disease (cSVD) constitutes a major risk factor for dementia. Monocytes play important roles in cerebrovascular disorders. Herein, we aimed to investigate the contribution of non-classical C-X3-C motif chemokine receptor (CX3CR)1 monocytes to cSVD pathobiology and therapy. To this end, we generated chimeric mice in which CX3CR1 in non-classical monocytes was either functional (CX3CR1^GFP/+^) or dysfunctional (CX3CR1^GFP/GFP^). cSVD was induced in mice via the micro-occlusion of cerebral arterioles, and novel immunomodulatory approaches targeting CX3CR1 monocyte production were used. Our findings demonstrate that CX3CR1^GFP/+^ monocytes transiently infiltrated the ipsilateral hippocampus and were recruited to the microinfarcts 7 days after cSVD, inversely associated with neuronal degeneration and blood-brain barrier (BBB) disruption. Dysfunctional CX3CR1^GFP/GFP^ monocytes failed to infiltrate the injured hippocampus and were associated with exacerbated microinfarctions and accelerated cognitive decline, accompanied with an impaired microvascular structure. Pharmacological stimulation of CX3CR1^GFP/+^ monocyte generation attenuated neuronal loss and improved cognitive functions by promoting microvascular function and preserving cerebral blood flow (CBF). These changes were associated with elevated levels of pro-angiogenic factors and matrix stabilizers in the blood circulation. The results indicate that non-classical CX3CR1 monocytes promote neurovascular repair after cSVD and constitute a promising target for the development of new therapies.

## Introduction

Cerebral small vessel disease (cSVD) encompasses several pathological and etiological divers microangiopathies that affect brain function.^1–5^ The neuroimaging hallmarks of cSVD comprise subcortical microinfarcts, lacunar infarcts, microbleeds, enlarged perivascular spaces, and white matter hyperintensities (WMH) associated with brain atrophy.^1,2^ Although heterogenous,^3^ common clinical manifestations exist, namely stroke-related symptoms, progressive cognitive decline, dementia, and psychiatric disorders.^4^ Obstruction of cerebral small penetrating arterioles leading to microinfarcts constitutes a major type of cSVD.^5^ These cerebral microangiopathies impair neurovascular functions, thus causing selective neuronal damage and neuroinflammation associated with impaired perfusion.^6–8^

Circulating monocytes play an essential role in maintaining neurovascular functions.^9–12^ Monocytes are mononuclear immune cells of hematopoietic origin that actively respond to sterile injuries.^13^ Their infiltration into the inflamed tissue is mediated by adhesion molecules, including intercellular adhesion molecule (ICAM)1.^14^ In rodents, monocytes are divided into 3 different subsets based on expression levels of the surface markers; lymphocyte antigen 6 complex-locus C (Ly6C), C-X3-C motif chemokine receptor (CX3CR)1 and the C-C chemokine receptor type (CCR)2.^15,16^ Classical Ly6C^high^CCR2^+^ monocytes infiltrate the injured tissue and contribute to the inflammatory response by differentiating into monocyte-derived macrophages (MDMs) cells expressing ionized calcium binding adaptor molecule (IBA)1 and cluster of differentiation (CD)68.^17^ Non-classical Ly6C^low^CX3CR1^+^ monocytes maintain vascular homeostasis.^11,18^ Upon injury, Ly6C^low^CX3CR1^+^ monocytes contribute to tissue repair via secretion of trophic factors and elimination of cell debris.^11,19^ Evidence suggests that CX3CR1^+^ monocytes infiltrate the injured brain, promoting neuroprotection upon excitotoxic insults,^20^ and differentiate into MDMs that stimulate neovascularization of damaged tissue via secretion of pro-angiogenic factors.^21,22^ Intermediate Ly6C^int^CX3CR1^int^CCR2^int^ monocytes constitute a distinct subset integrating the characteristics of classical and non-classical monocytes.^23^ CX3CR1 regulates monocyte migration,^24,25^ and its depletion attenuates the latter under pathological conditions^26^ and compromises their survival,^27^ thus deregulating tissue neovascularization.^21^ Moreover, triggering receptor expressed on myeloid cells (TREM)2 regulates migration and survival of MDMs,^28^ which release insulin-like growth factor (IGF)1 to promotes neurovascular protection.^29,30^ Interestingly, classical monocytes can give rise to non-classical subset via activation of the transcription factor nuclear receptor subfamily 4 group A member (Nr4a)1.^13,31–33^

Our study aims to elucidate the role of non-classical monocytes in cSVD pathobiology and therapy. For this purpose, we generated chimeric mice in which CX3CR1 is either expressed in bone marrow-derived monocytes (CX3CR1^GFP/+^; functional cells) or depleted (CX3CR1^GFP/GFP^; dysfunctional cells), combined with immunomodulatory approaches to stimulate non-classical monocyte generation.

## Materials and methods

### Animal experiments

We used 3–5 months old male C57Bl6/J wildtype (WT), CX3CR1^GFP/+^ and CX3CR1^GFP/GFP^ mice. In heterozygous CX3CR1^GFP/+^ mice [B6.129P2(Cg)-Cx3cr1^tm1Litt/J^], an enhanced green fluorescent protein (GFP) sequence replaces the first 390 bp of coding exon 2 in 1 allele of CX3CR1 gene allowing tracking of functional monocytes (CX3CR1^GFP/+^ monocytes). In homozygous CX3CR1^GFP/GFP^ mice, the coding exons of both alleles are replaced by GFP leading to CX3CR1 depletion (dysfunctional CX3CR1^GFP/GFP^ monocytes). CX3CR1^GFP/+^ and CX3CR1^GFP/GFP^ mice were used as donors to generate chimeric mice. Subgroups of CX3CR1^GFP/+^ and WT mice were used to evaluate the infiltration of non-classical monocytes and cerebral blood flow (CBF), respectively. Mice were housed under standard laboratory conditions and were provided with standard chow and water *ad libitum*. Mice were randomized for the different experiments, and experimenters were completely blind to genotype/experimental condition. Animal procedures were performed according to the Canadian Council on Animal Care guidelines, as implemented by *Comité de Protection des Animaux de l'Université Laval-3* (CPAUL-3; Protocol # 2020-387). Animal studies were reported according to ARRIVE 2.0 guidelines.

### Generation of chimeric mice

WT recipient mice received 2x intraperitoneal injections (every 8 h) of Busulfan (Otsuka America Pharmaceutical; 10 mg/kg) per day for 4 days followed by 1× intraperitoneal injection of cyclophosphamide (100 mg/kg) per day for 2 days. Mice were kept in sterile cages and were given previously irradiated food and received continuous antibiotics. At the day of transplantation, femurs of CX3CR1^GFP/+^ and CX3CR1^GFP/GFP^ donor mice were flushed to extract bone marrow cells using Dulbecco's Phosphate Buffered Saline (DPBS) complemented with 5% fetal bovine serum (FBS). Cell extracts were filtered on a 40 *µ*m nylon filter. Cell pellets were collected in DPBS, centrifuged and re-suspended in DPBS. The following chimeric mice were generated; CX3CR1^GFP/+^ → WT chimeric mice and CX3CR1^GFP/GFP^ → WT chimeric mice (KO chimeric mice). Bone marrow reconstitution was confirmed 8 weeks later by evaluating GFP expression in Ly6C^+^ cells using flow cytometry, and chimerism average was established around 60%.^6,34,35^

### Muramyl dipeptide (MDP) regimen

MDP is a synthetic immunoreactive peptide that promotes conversion of classical monocytes into non-classical subset through Nr4a1 activation.^32^ Five mg of MDP (TLRL MDP, Cedarlane) were resuspended in 500 µl of endotoxin-free water, and intraperitoneally administered (10 mg/kg, 1x per day for 3 days) into mice to prime monocytes prior to cSVD, followed by a recall injection 7 days later. CX3CR1^GFP/+^ → WT mice were divided into non-treated control (C chimeric mice) or MDP-treated (MDP chimeric mice) groups. Subgroup of WT mice and CX3CR1^GFP/+^ mice treated with MDP or non-treated (Control, C) were used as well.

### Multifocal cerebral micro-occlusion

cSVD was induced through the micro-occlusion of penetrating cerebral arterioles.^6^ Mice were anesthetized under 1.5% isoflurane in 1.5 l/min (95% O_2_) and body temperature was maintained between 36–37°C using a feedback-controlled heating system (Harvard Apparatus, QC, Canada). A midline neck incision was performed to expose the left common carotid artery (CCA) under a surgical microscope (Leica Microsystems, ON, Canada). The external carotid artery (ECA) and pterygopalatine artery (PPA) were temporarily blocked using microvascular clips. Next, 2500 sterilized FITC-labeled 20 *µ*m microspheres (Polysciences Inc., PA, USA) suspended in a 100 µl of PBS were injected into the CCA using a 33 G hypodermic needle (TSK Laboratory International, BC, Canada).^6,7^ The needle was next gently removed and bleeding was stopped by applying pressure using bioabsorbable Gelfoam (Pfizer, NY, USA), and ECA and PPA were unblocked. Survival rate was tracked.

### Flow cytometry experiments

Blood samples were collected from the submandibular vein into ethylene-diamine-tetra-acetic acid (EDTA) coated vials (Sarstedt, Montréal, QC, Canada). Briefly, 60 μl of total blood was incubated at room temperature (RT) for 20 min in 1 ml of ACK (ammonium, chloride, potassium) lysing buffer to remove erythrocytes. Samples were washed with 3 ml of DPBS and centrifuge at 400×g for 8 min at 4°C. Supernatant was removed, and cells were resuspended with 100 µl of blockage solution, 1 µl CD16/CD32 antibody (BD bioscience) diluted in 100 μl of DPBS per tube and incubated for 10 min on ice. Next, 100 µl of antibody mix was added in each tube for 30 min on ice (Supplementary Table 1). Remaining cells were then washed with 3 ml DPBS, centrifuged for 8 min at 450×g, resuspended in 300 µl of DPBS, and complemented with 50 µl of 123count eBeads™ Counting Beads (Invitrogen). Samples were processed using LSR II flow cytometer and data acquired using BD FACS Diva software (Version 6.1.2, BD Bioscience) and analyzed using FlowJo software v10 (Tree Star; Ashland, OR, USA).^6,36^

### Neurobehavioral analysis

Novel object recognition (NOR) test was used to assess recognition memory.^6,34^ In an open arena mice freely explored 2 identical objects for 10 min in the acquisition phase, placed back in their home cage for 1 h and tested in the 10 min retention phase during which one object was replaced. Mice normally spend more time exploring novel objects during retention phase.^37,38^ Trials were recorded by a camera placed above the arena and a blind experimenter assessed time spent on each object on the collected videos. The arena and objects were cleaned with 70% ethanol (EtOH). The task was performed at baseline and 7 days post-cSVD. A discrimination index was calculated as follows: time novel object/(time novel object - time familiar object). An index =0.5 means an equal exploration of the 2 objects, an index > 0.5 means a preference for the novel object, and <0.5 means a preference for the familiar object.

Elevated Plus Maze (EPM) test was used to evaluate anxiety-related behavior.^39^ The apparatus is a “+”-shaped maze, elevated above the floor with two oppositely open-arms and two closed-arms. Trials were recorded by a camera placed above the arena and a blind experimenter scored the time spent by each animal in the open- or closed-arms on the collected videos. The maze was systemically cleaned using 70% EtOH. The test was performed at baseline and 7 days post-cSVD. A reduced time spent in the open arms translates an anxiety-like behavior.^39^

### Laser speckle contrast imaging (LSCI) analysis

LSCI was used to analyze CBF at baseline, 24 h, 3 and 7 days after cSVD in WT C and MDP mice. Mice were anesthetized using 1.5% isoflurane in 1.5 l/min (95% O_2_). Prior to surgery, the head was shaved, lidocaine/bupivacaine solution applied on the incision site (100 µl) and the ears (50 µl/ear), and the skin disinfected. Mice were placed on a stereotaxic frame (RWD Life Science Inc., CA, USA), the skull was exposed by removing the skin using fine-tip forceps, and relative CBF was measured using 2 D laser Speckle blood flow imager (OMEGAZONE OZ-3, OMEGAWAVE, INC.). The OZ-3 system is equipped with a visible and near infra-red (NIR) CCD camera, which allows showing real images continuously while comparing the color difference between real and blood flow images. Furthermore, the system is equipped with a measurement and analysis software that allows simultaneous quantification of the relative CBF values in different regions of interest (ROI). The mean of relative CBF of each hemisphere in similar ROI was quantified, and a ratio of ipsilateral/contralateral values were computed. A ratio of 1 indicates that similar CBF values in both hemispheres, and a ratio below 1 suggests a hypoperfusion in the ipsilateral hemisphere, while a ratio above 1 outlines a hyperperfusion.

### Tissue sample preparation

WT and CX3CR1^GFP/+^ → WT chimeric mice were euthanized at day 3, 7 and 1 month after cSVD. C, MDP, and KO chimeric mice were euthanized at day 7. WT or CX3CR1^GFP/+^ C and MDP mice were euthanized 3 or 7 days post-cSVD. Mice were anesthetized with ketamine/xylazine (90 mg/ml; 10 mg/ml) and euthanized via intracardiac perfusion with phosphate buffer saline (PBS), followed for some by 4% paraformaldehyde (PFA). PFA-perfused brains were postfixed in 4% PFA for 24 h and transferred into PBS containing 20% sucrose for 24 h and PBS-perfused brains were frozen on dry ice and stored at −80°C until use. PFA-fixed brains were cut into 25 μm coronal sections on microtome (Leica Biosystems, ON, Canada), and serial sections were collected in 12 well-plates containing an antifreeze solution (30% glycerol, 30% ethylene glycol in 0.9% sodium chloride (NaCl), phosphate buffer (PB)) and kept at −20°C for further use. PBS-perfused brains were cut at 20 *µ*m on a cryostat, directly mounted onto Superfrost® Plus slides and stored at −80°C until use.

### Fluoro-Jade B (FJB) staining

Degenerating neurons were labeled using FJB staining. Brain sections were washed with potassium phosphate buffer saline (KPBS) 3× 10 min, mounted onto Superfrost® Plus slides and dried overnight. Next day, mounted sections were fixed with 4% PFA for 30 min, rinsed 2x with KPBS for 5 min and processed through a cycle of dehydration/rehydration in EtOH (3 min in 50%, 1 min in 70%, 3 min in 100%, 1 min in 70%, 1 min in 50% and 1 min in distilled water). Sections were treated for 10 min with 0.06% potassium permanganate (MP Biomedicals, Santa Ana, CA, USA), rinsed for 7 min with distilled water, and incubated in 0.2% FJB solution (EMD Millipore, Etobicoke, ON, Canada) containing 0.1% acetic acid and 0.1% 4′,6-diamidino-2-phenylindole (DAPI) in milliQ water for 10 min. Sections were rinsed in milliQ water and dried overnight, then immersed 3 × 2 min in Xylene, and cover-slipped with Dibutylphthalate Polystyrene Xylene (DPX). FJB^+^ cell density was assessed using unbiased computer-assisted stereological software (Stereologer; SRC Biosciences, FL, USA).^40^

### Immunohistochemical analysis

To assess the brain infiltration of endogenous immunoglobulin G (IgG), free-floating brain sections were rinsed 3× with KPBS, and then incubated at RT in a blocking/permeabilization solution containing 4% normal goat serum (NGS), 1% bovine serum albumin (BSA), 1% Triton X-100 in KBPS for 45 min. Sections were next incubated overnight with a biotinylated goat anti-mouse Immunoglobulin G (IgG; H + L, 1:1000, Vectorlab, BA-9200). Sections were rinsed 3× with KPBS and incubated for 30 min at RT with Avidin-Biotin Peroxidase Complex (ABC, Vectastain Elite Kit Standard). Next, sections were rinsed 3x with KPBS and incubated with 3,3ʹ-diaminobenzidine tetrahydrochloride (DAB; Sigma-Aldrich), washed 3x with KPBS, mounted onto Superfrost® Plus slide and dried overnight at RT. Next day, sections on slides were dehydrated via immersion in an increased concentration of EtOH solution (50%, 70%, 75%, 95% and 100%), and immersed in Xylene solution 2× for 3 min. The mounted slides were then cover-slipped with DPX. The slides were scanned, and the area covered by DAB was analyzed using ImageJ software, as previously described.^6^

### Immunofluorescence analysis

Free-floating brain sections were processed as above while fresh frozen samples underwent first an incubation of 5 min in methanol at −20°C. Sections were incubated with primary antibodies (Supplementary Table 2) diluted in blocking/permeabilization solution overnight at 4°C. Next day, sections were rinsed 3× with KPBS, and incubated for 2 h at RT with the adequate Cy3 or Cy5-conjugated secondary antibody (Supplementary Table 2). Sections were rinsed 2× with KPBS and incubated with DAPI (1:10 000, Invitrogen) for 5 min. Brain sections were mounted onto Superfrost® Plus slides and cover-slipped with Fluoromount-G® anti-fade medium (Sigma-Aldrich). Epifluorescence images were taken using an Axio Observer microscope equipped with an optical sectioning module (Apotome.2) and Axiocam 503 monochrome camera, and processed in ZEN Imaging Software (Carl Zeiss Canada, Toronto, ON, Canada). Density of IBA1, CD45, neuron-specific class III β tubulin (TUJ1), neuronal nuclear protein (NeuN), doublecortin (DCX), CD31, ICAM1, glial fibrillary protein (GFAP) and transmembrane protein (TMEM119) immunolabeling and microtubule-associated protein (MAP2) depleted volume were assessed using unbiased computer-assisted stereological software (Stereologer; SRC Biosciences, FL, USA).^6^ CD68 intensity, claudin 5 density, aquaporin (AQP)4 coverage and CD31 vessel diameter were assessed using ImageJ software.

### RNAscope® multiplex fluorescent in situ hybridization (FISH)

Free-floating brain sections were mounted onto Superfrost® Plus slides and dried for 1 h at −20°C before being backed for 30 min at 60°C, followed by 15 min bath in 4% PFA. Mounted sections were dehydrated using EtOH and incubated in hydrogen peroxidase (H_2_O_2_). Following retrieval, mounted sections were immersed in 100% EtOH and a hydrophobic barrier was drawn. RNAscope® fluorescent multiplex reagent kit was used following manufacturer’s recommendations (Advanced Cell Diagnostics, Newark, CA, USA). Five drops of Protease III were added to cover the sections, and incubated 40 min at RT. Following a series of wash with 0.1 M PBS, 4 drops of RNAscope® Probe-TREM2-C2 and RNAscope® Probe-IGF1-C3 (Advanced Cell Diagnostics) were added to each section, slides were placed into a rack, and inserted in HybEZ^TM^ Oven humidity control tray for 2 h at 40°C. The tray was removed, one slide at a time, excess of liquid was removed, and slides were washed with 1× wash buffer. Four drops of Amp-1 were added to entirely cover each section, followed by incubation in the HybEZ^TM^ Oven for 30 min at 40°C. This process was repeated with Amp-2 for 30 min and Amp-3 for 15 min. TREM2 and IGF1 mRNAs were simultaneously detected using Opal^TM^ 520 (1:700) and Opal^TM^ 690 (1:700; AKOYA Biosciences®, MA, USA), respectively. Finally, 4 drops of DAPI were added to each section and kept for 30 s. Slides were mounted with 110 μl of Fluoromount-G®, cover-slipped, and stored at 4°C in the dark until analysis under microscope (Carl Zeiss Canada).

### Proteome Profiler^TM^ mouse angiogenesis array

CX3CR1^GFP/+^ C and MDP mice were transcardially perfused 3 days post-cSVD. Blood was collected and allowed to clot in Eppendorf tubes for 2 h at RT and centrifuged at 2000×g for 20 min. Supernatant was collected and processed in a membrane-based Proteome Profiler**^TM^** mouse angiogenesis array kit (R&D, ARY015), following manufacturer’s recommendations. Membranes were digitized in Biorad chemidoc XRS+ (Biorad, Montreal, QC, Canada) and dots were densitometrically analyzed using ImageJ software and expressed as relative values.

### Statistical analysis

Data are presented as boxplot with min/max whiskers or mean ± standard deviation (SD). Boxplots and descriptive statistics were used to assume data distribution normality. When normality assumption was respected, unpaired two-tailed *t*-test for comparison between two groups and one-way analysis of variance (ANOVA) followed by Tukey’s post-hoc test for multiple comparison were used. Two-way ANOVA followed by Tukey’s post-hoc test was used to assess interaction between experimental conditions and hemispheres. When normality assumption was violated, Kruskal-Wallis test followed by Dunn’s post-hoc test for multiple comparison were used. Kaplan-Meier estimate showing survival rate and log-rank test comparing two groups were used. P-value *<*0.05 was considered statistically significant (Tukey’s test, 95% CI for the difference; Dunn’s test, mean rank difference (M_rank_) = difference of the mean rank scores of given two groups ranked in Kruskal-Wallis test). Statistical analyses were carried out using GraphPad Prism Version 9.0 for OS X (GraphPad Software).

## Results

### cSVD induces neuronal degeneration associated with vascular permeability and recruitment of non-classical monocytes

**Figure 1.**
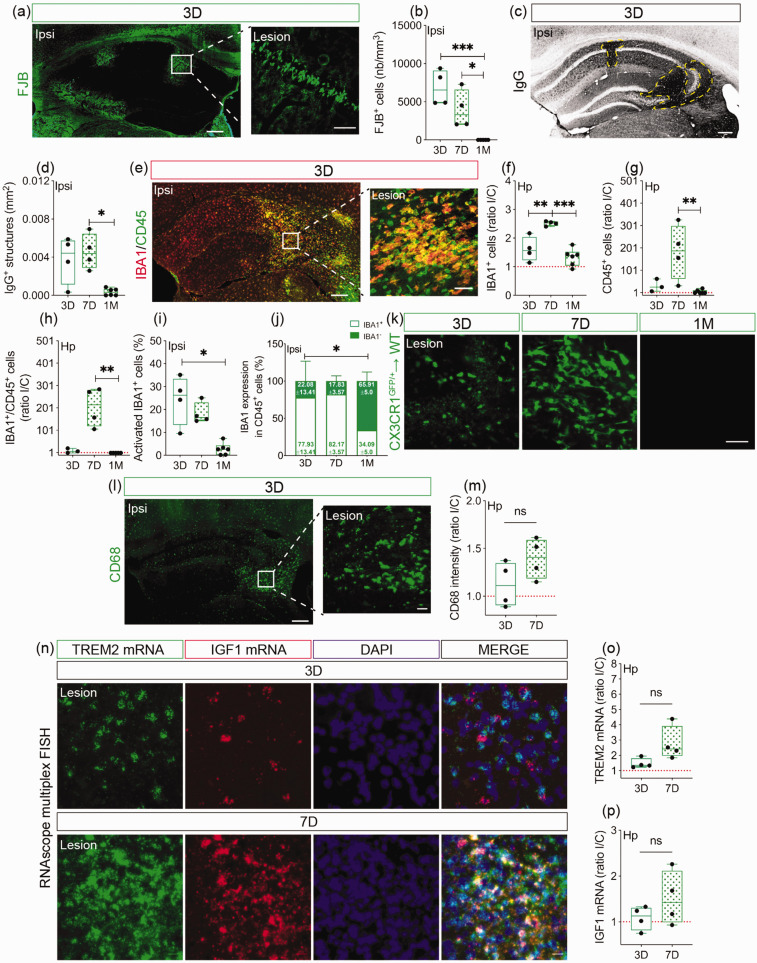
Temporal analysis of structural damage and neuroinflammation in the hippocampus after cSVD. (a) Representative images of FJB^+^ degenerating neurons in the ipsilateral hippocampus and a close caption of FJB^+^ cells in CA1 region 3 days after cSVD. (b) Stereological analysis of the absolute density of FJB^+^ degenerating neurons in the ipsilateral hippocampus. (c) Representative images of blood-borne IgG immunolabeling (yellow dashed line) in the ipsilateral hippocampus after cSVD. (d) Analysis of the area occupied by extravasating IgG in the ipsilateral hippocampus. (e) Representative images of IBA1 and CD45 co-immunolabeling in the ipsilateral hippocampus and a close caption in the lesion site 3 days after cSVD. Stereological analysis of (f) IBA1, (g) CD45 and (h) IBA1/CD45 density in the hippocampus, shown as ipsilateral/contralateral (I/C) ratio. (i) Temporal analysis of the percentage of IBA1^+^ cells expressing CD45 (reactive microglia) in the ipsilateral hippocampus. (j) Analysis of the temporal changes in IBA1 expression in overall CD45^+^ cell population in the ipsilateral hippocampus (white box = percentage of CD45^+^ cells expressing IBA1; Continued.green box = percentage of CD45^+^ cells not expressing IBA1). (k) Representative fluorescent images of non-classical monocytes (CX3CR1^GFP/+^ cells) recruited to the ipsilateral hippocampus of CX3CR1^GFP/+^ → WT chimeric mice 3, 7 days and 1 month after cSVD. (l) Representative fluorescent images of CD68^+^ cells (phagocyting cells) in the ipsilateral hippocampus and a close caption in the lesion site 3 days after cSVD. (m) Analysis of CD68^+^ expression (fluorescent intensity) at day 3 and 7 after cSVD, shown as I/C ratio. (n) Representative fluorescent images of TREM2 and IGF1 mRNA expression in the ipsilateral hippocampus at days 3 and 7 after cSVD. (o) Analysis of TREM2 and IGF1 mRNA temporal expression at day 3 and 7 after cSVD, shown as I/C ratio. Data are boxplot with min/max and mean ± SD (white box) (j) (n = 3–6 animals/group). I/C ratio = 1 indicates similar pattern in ipsilateral and contralateral hemispheres. *P < 0.05/^**^P < 0.01/^***^P < 0.001 compared to 3 or 7 days after cSVD (one-way ANOVA or Kruskal-Wallis test). Statistical summary is provided in (Supplementary Material 2). Scale bar = 200 µm (a, c, e, l); 50 µm (close captions; a, e, l, k, n). D, days; M, month.

The hippocampus, which plays a major role in memory and anxiety that are impaired in cSVD, is highly vulnerable to hypoxic and ischemic injuries.^6,39,41^ Yet, hippocampal pathobiology after cSVD remains not fully explored. Structural damage and infiltration of CX3CR1^GFP/+^ monocytes were evaluated in the ipsilateral hippocampus at day 3, 7 and 1 month after cSVD. Analysis of FJB staining (Figure 1(a)) indicated that the density of FJB^+^ cells (degenerating neurons) peaked at day 3 and gradually decreased at day 7, while FJB^+^ cells were absent at 1 month (3 D vs 1 M, CI [3401, 10259], P = 0.0007; 7 D vs 1 M, CI [554.7, 7413], P = 0.0241) (Figure 1(b)). Next, blood-brain barrier (BBB) permeability was assessed by analyzing IgG extravasation into the ipsilateral hippocampus (Figure 1(c), yellow dashed line). IgG^+^ structures increased 3 days after cSVD, remained elevated at day 7, and decreased at 1 month (7 D vs 1 M, M_rank_ = 6.75, P = 0.037) (Figure 1(d)). The dynamics of microglia/MDMs, which are activated upon ischemia^42,43^ were evaluated by immunolabeling IBA1 and CD45 (Figure 1(e)). Expression of IBA1 (3 D vs 7 D, CI [−1.484, −0.3214], P = 0.0039; 7 D vs 1 M, CI [0.6504, 1.711], P = 0.0002) (Figure 1(f)), CD45 (7 D vs 1 M, M_rank_=7.417, P = 0.009) (Figure 1(g)) and IBA1/CD45 colocalization (7 D vs 1 M, M_rank_ = 7, P = 0.006) (Figure (h)) increased in the ipsilateral hemisphere at day 7 after cSVD compared to day 3 and 1 month. Density of activated microglia/MDMs (IBA1^+^/CD45^+^) increased 3 days after cSVD, and decreased overtime, to reach basal levels at 1 month (3 D vs 1 M, M_rank_ = 7.75, P = 0.0123) (Figure 1(i)). Interestingly, CD45^+^ cells that do not express IBA1 (CD45^+^/IBA1^−^), outlining a peripheral immune cell origin, were present at the lesion site, representing 22.08 ± 13.41% and 17.83 ± 3.57% of CD45^+^ cell population at day 3 and 7 respectively, and 65.91 ± 5% at 1 month (3 D vs 1 M, M_rank_ = 6.3, P = 0.0477) (Figure 1(j)). Using CX3CR1^GFP/+^ → WT chimeric mice, we confirmed CX3CR1^GFP/+^ monocyte recruitment at the lesion site at day 3 and 7 after cSVD, and absence at 1 month (Figure 1(k)). Infiltrating monocytes were further characterized by immunolabeling CD68 (Figure 1(l)), showing a tendency to increase in the ipsilateral hippocampus at day 7, the time point at which CX3CR1^GFP/+^ monocyte infiltration peaked (CI [−0.1138, 0.6586], P = 0.1351; 3 D = 1.121 ± 0.2352; 7 D = 1.394 ± 0.2105) (Figure 1(m)). Next, we analyzed TREM2 and IGF1 spatiotemporal mRNA expression using RNAscope® multiplex FISH (Figure 1(n)). TREM2 mRNA levels tended to increase in the ipsilateral hippocampus at day 3, and more importantly at day 7 (CI [−0.1246, 2.725], P = 0.067; 3 D = 1.468 ± 0.3279; 7 D = 2.768 ± 1.117) (Figure 1(o)), while IGF1 mRNA levels slightly augmented at day 7 compared to day 3 (CI [−0.3628, 1.213], P = 0.2349; 3 D = 1.085 ± 0.2585; 7 D = 1.51 ± 0.5897) (Figure 1(p)). TREM2 and IGF1 mRNA expression increased at the lesion site at day 7 after cSVD, a time point associated with important recruitment of CX3CR1^GFP/+^ monocytes. Our results indicate that cSVD induces neurodegenerative responses in the hippocampus associated with BBB breakdown and transient recruitment of CX3CR1 monocytes.

### Modulating CX3CR1 function affects the dynamics of non-classical monocytes upon cSVD

**Figure 2.**
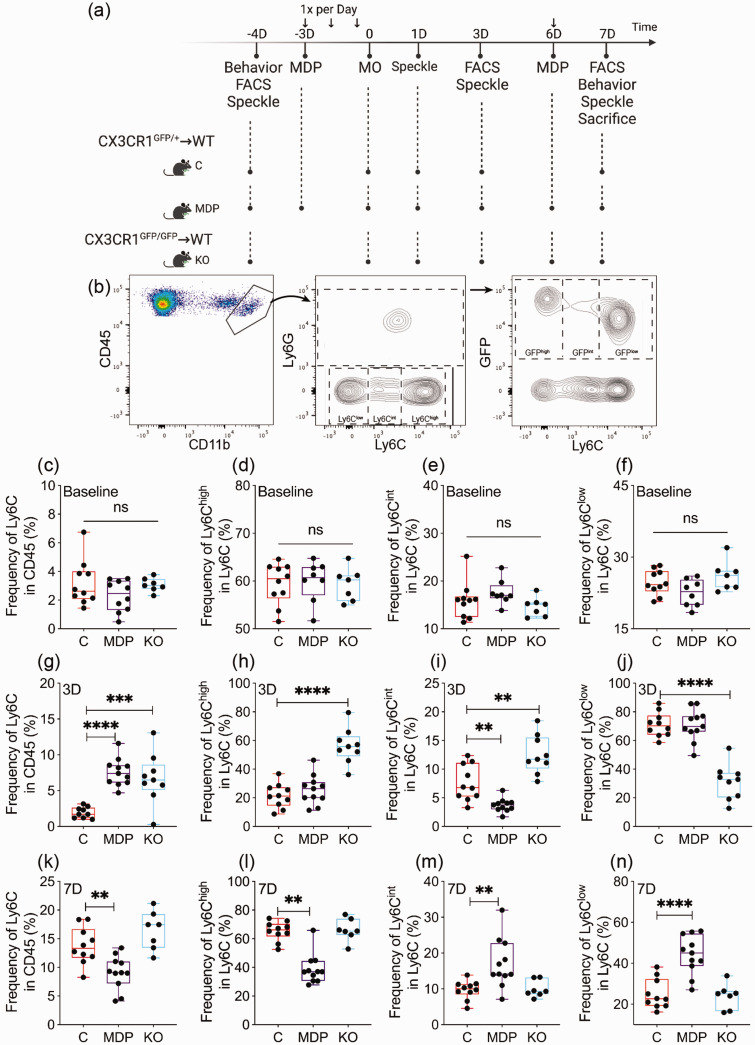
Dynamics of monocytes in the blood circulation is affected by the manipulation of CX3CR1 subset and cSVD. (a) A scheme illustrating study’s experimental design. Created with BioRender.com. (b) Gating strategy used to discriminate circulating monocytes (CD11b^+^Ly6C^+^) in leukocytes (CD45^+^) in the blood circulation, distribution of classical monocytes (Ly6C^high^CX3CR1^−^), intermediate monocytes (Ly6C^int^CX3CR1^+^) and non-classical monocytes (Ly6C^low^CX3CR1^+^), as well as GFP expression in Ly6C^+^ monocytes. Flow cytometry analysis of (c) total monocytes, (d) classical (Ly6C^high^), I intermediate (Ly6C^int^) and (f) non-classical (Ly6C^low^CX3CR1^+^) frequencies at baseline prior to cSVD. Flow cytometry analysis of (g) total monocytes, (h) classical (Ly6C^high^), (i) intermediate (Ly6C^int^) and (j) non-classical (Ly6C^low^CX3CR1^+^) frequencies 3 days after cSVD. Flow cytometry analysis of (k) total monocytes, (l) classical (Ly6C^high^), (m) intermediate (Ly6C^int^) and (n) non-classical (Ly6C^low^CX3CR1^+^) frequencies 7 days after cSVD. Data are boxplot with min/max (n = 7–11 animals/group). **P < 0.01/***P < 0.001/****P < 0.0001 compared to C chimeric mice (one-way ANOVA or Kruskal-Wallis test). Statistical summary is provided in (Supplementary Material 2). D, days.

Monocyte subsets are differentially regulated upon cSVD.^6^ Here, we used chimeric mice in which bone marrow-derived CX3CR1^+^ monocytes are either functional (CX3CR1^GFP/+^) or dysfunctional (CX3CR1^GFP/GFP^). cSVD was induced in C, MDP, and KO chimeric mice, as previously described.^6^ Flow cytometry analysis was performed at baseline, 3 and 7 days after cSVD (Figure 2(a)). Using a gating strategy to identify circulating monocyte subsets based on Ly6C expression, we validated GFP^+^ CX3CR1^+^ monocyte presence in Ly6C^low^ population in chimeric mice (Figure 2(b)). At baseline, frequencies of total monocytes (P = 0.2302) (Figure 2(c)) as well as Ly6C^high^ (P = 0.9440) (Figure 2(d)), Ly6C^int^ (P = 0.1967) (Figure 2(e)), and Ly6C^low^ (CX3CR1^+^) subsets (P = 0.0635) (Figure 2(f)) were similar in all groups. However, total monocyte frequency increased in KO and MDP chimeric mice compared to C chimeric mice at day 3 (C vs MDP, CI [−8.237, −3.059], P < 0.0001; C vs KO, CI [−7.485, −2.055], P = 0.0005) (Figure 2(g)). Frequency of Ly6C^high^ subset increased in KO chimeric mice compared to C chimeric mice (C vs KO, CI [−46.62, −23.17], P < 0.0001) (Figure 2(h)). Ly6C^int^ subset frequency increased in KO chimeric mice while it decreased in MDP chimeric mice compared to C chimeric mice (C vs MDP, CI [1.002, 6.8], P = 0.0068; C vs KO, CI [−8.008, −1.91], P = 0.0011) (Figure 2(i)). Ly6C^low^ subset frequency decreased in KO chimeric mice compared to C chimeric mice (C vs KO, CI [27.71, 52.01], P < 0.0001) (Figure 2(j)). MDP reduced total monocyte frequency at day 7 (C vs MDP, CI [1.267, 8.107], P = 0.006) (Figure 2(k)), and decreased Ly6C^high^ subset frequency (C vs MDP, M_rank_ = 12.58, P = 0.0014) (Figure 2(l)) while increasing that of Ly6C^int^ (C vs MDP, CI [−12.55, −2.167], P = 0.0045) (Figure 2(m)) and Ly6C^low^ subsets (C vs MDP, CI [−27.89, −10.4], P < 0.0001) (Figure 2(n)) compared to C chimeric mice, indicating that Ly6C^high^ monocytes are actively switching into Ly6C^low^CX3CR1^+^ subset. Circulating neutrophils increased in KO chimeric mice 3 and 7 days after cSVD compared to C chimeric mice (3 D, CI [−22.28, −6.981], P = 0.0003; 7 D, CI [−2.391, −0.02828], P = 0.044) (Supplementary Figure 1). Our results suggest that monocytes are differentially regulated after cSVD dependently upon CX3CR1 expression.

### Neuronal loss and cognitive deficits upon cSVD are affected by CX3CR1 function in monocytes

**Figure 3.**
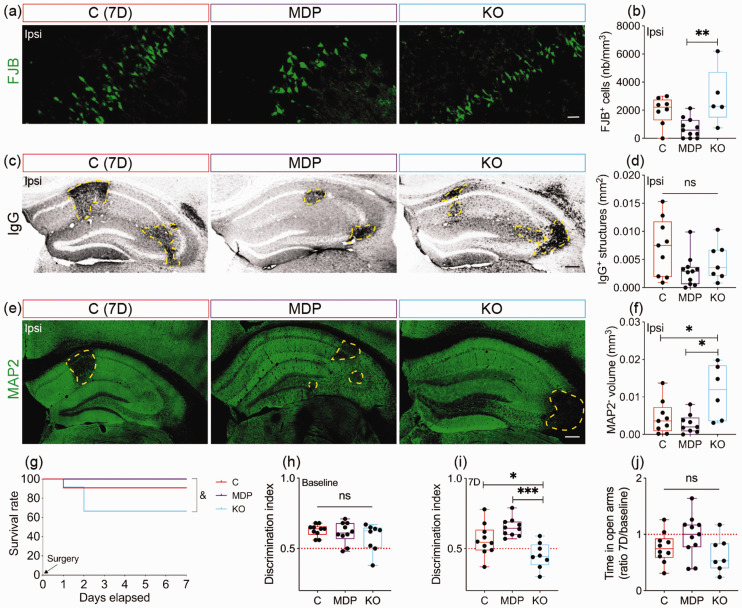
CX3CR1 expression manipulation in monocyte modulates structural damage and cognitive functions after cSVD. (a) Representative fluorescent images of FJB^+^ degenerating cells in the ipsilateral hippocampus of C chimeric mice, MDP chimeric mice and KO chimeric mice 7 days after cSVD. (b) Stereological analysis of the absolute density of FJB^+^ degenerating neurons in the ipsilateral hippocampus. (c) Representative images of blood-borne IgG immunolabeling (yellow dashed lines) in the ipsilateral hippocampus of C, MDP and KO chimeric mice 7 days after cSVD. (d) Analysis of the area occupied by extravasated IgG in the ipsilateral hippocampuI (e) Representative fluorescent images of MAP2 immunolabeling in the ipsilateral hippocampus of C, MDP and KO chimeric mice 7 days post-cSVD. (f) Stereological analysis of the volume of MAP2 depleted region (yellow dashed lines) in the ipsilateral hippocampus. (g) Kaplan-Meier estimate of the survival rate until day 7 post-cSVD. Analysis of NOR test performed at (h) baseline and (i) 7 days after cSVD. (j) Analysis of the time spent in the open arms in the EPM test at day 7 corrected to baseline. Data are boxplot with min/max (n = 5–11 animals/group). Discrimination index = 0.5 indicates that mice equally explored familiar and novel object. *P < 0.05/**P < 0.01/***P < 0.001 compared to C or MDP chimeric mice (one-way ANOVA); ^&^P < 0.05 KO chimeric mice compared to MDP chimeric mice (log-rank test (g)). Statistical summary is provided in (Supplementary Material 2). Scale bar = 20 µm (a); 200 µm (c, e). D, days.

Depletion of non-classical monocytes exacerbates neuronal death upon sterile excitotoxic insult.^20^ We aimed here to evaluate the impact of manipulating CX3CR1 expression in monocytes on neuronal damage after cSVD. Density of FJB^+^ cells was reduced in the ipsilateral hippocampus of MDP chimeric mice compared to KO chimeric mice 7 days after cSVD (MDP vs KO, CI [−3802, −644.2], P = 0.0052) (Figure 3(a) and (b)). This was accompanied with a mild reduction in IgG extravasation into the ipsilateral hippocampus of MDP chimeric mice (P = 0.0731; C = 0.007178 ± 0.005117; MDP = 0.003025 ± 0.002753; KO = 0.004632 ± 0.003337) (Figure 3(c) and (d)). MAP2 depletion volume (i.e. neuronal loss) in the ipsilateral hippocampus (Figure 3(e)), was elevated in KO chimeric mice compared to C chimeric mice and to a greater extent compared to MDP chimeric mice (C vs KO, CI [−13292400, −525053], P = 0.0325; MDP vs KO, CI [−14726213, −1643576], P = 0.0129) (Figure 3(f)). MDP chimeric mice exhibited a higher survival rate compared to KO chimeric mice (MDP vs KO, log-rank test, P = 0.04) (Figure 3(g)). CX3CR1 manipulation in non-classical monocytes did not affect neurorestoration (NeuN, P = 0.7031; DCX, P = 0.2349; TUJ1, P = 0.4106) (Supplementary Figure 2). Assessment of cognitive functions indicated that at baseline, recognition memory was not affected in all groups (P = 0.3366) (Figure 3(h)). However, MDP chimeric mice exhibited improved memory, translated by a better discrimination of the novel object compared to KO chimeric mice, which performed worse compared to C chimeric mice (C vs KO, CI [0.0002665, 0.2192], P = 0.0494; MDP vs KO, CI [0.09027, 0.3092, P = 0.0003) (Figure 3(i)) 7 days after cSVD. Furthermore, time spent by MDP chimeric mice in the EPM open arms at day 7 was slightly restored (Figure 3(j)), outlining a potential alleviation of the anxiety-related behavior (P = 0.1116; C = 0.764 ± 0.2702; MDP = 0.972 ± 0.3712; KO =0.6429 ± 0.3153). Our results suggest that CX3CR1^GFP/+^ monocytes alleviate neuronal loss after in the hippocampus in cSVD and decelerate cognitive decline.

### MDP accelerates non-classical monocyte infiltration into the brain upon cSVD

**Figure 4.**
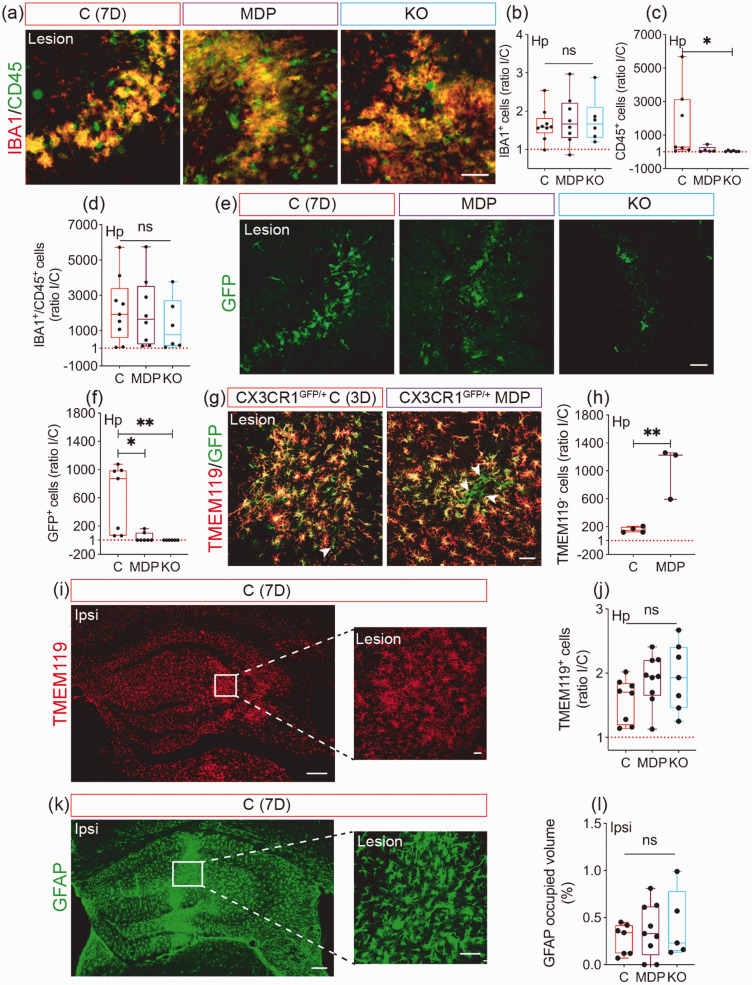
Infiltration of CX3CR1^GFP/+^ monocytes into the ipsilateral hippocampus after cSVD is accelerated upon MDP administration. (a) Representative fluorescent images of IBA1 and CD45 co-immunolabeling in the ipsilateral hippocampus 7 days post-cSVD. Stereological analysis of (b) IBA1^+^, (c) CD45^+^ and (d) IBA1^+^/CD45^+^ cell densities ratio in the hippocampus, shown as ipsilateral/contralateral (I/C) rIo. (e) Representative fluorescent images of GFP^+^ cells (CX3CR1^+^ monocytes) in the hippocampus of C, MDP and KO chimeric mice outlining the differential infiltration of cells 7 days post-cSVD. (f) Stereological analysis of GFP^+^ cell density in the hippocampus, shown as I/C ratio. (g) Representative fluorescent images of GFP^+^ CX3CR1^+^ cells immunolabeled with TMEM119 3 days after cSVD in the ipsilateral hippocampus of CX3CR1^GFP/+^ C and MDP mice. (h) Stereological analysis of GFP^+^ CX3CR1^+^ TMEM119^−^ cell density (infiltrating MDMs) in the hippocampus 3 days after cSVD, shown as I/C ratio. (i) Representative fluorescent images of TMEM119 immunolabeling in the ipsilateral hippocampus and a close caption in the lesion site 7 days post-cSVD. (j) Stereological analysis of TMEM119^+^ cell density (resident microglia) in the hippocampus 7 days after cSVD, shown as I/C ratio. (k) Representative fluorescent images of GFAP immunolabeling in the ipsilateral hippocampus and close caption in the lesion site 7 days post-cSVD. (l) Stereological analysis of the volume occupied by reactive GFAP^+^ cells in the ipsilateral hippocampus 7 days after cSVD. Data are boxplot with min/max (n = 3–9 animals/group). I/C ratio = 1 indicates similar pattern in ipsilateral and contralateral hemispheres. *P < 0.05/**P < 0.01 compared to C chimeric mice (unpaired two-tailed *t*-test, one-way ANOVA or Kruskal-Wallis test). Statistical summary is provided in (Supplementary Material 2). Scale bar = 200 µm (i, k); 50 µm (close captions; a, e, g, i, k). D, days.

We aimed here to investigate the impact of manipulating CX3CR1 function in monocytes on microglial activation and immune cell infiltration. Analysis of IBA1 and CD45 reactivity (Figure 4(a)) showed that IBA1^+^ cell density similarly increased in the ipsilateral hemisphere of all groups (P = 0.8643; C = 1.64 ± 0.4314; MDP = 1.771 ± 0.657; KO = 1.768 ± 0.599) (Figure 4(b)), whereas CD45^+^ cell density tended to decrease in MDP and to significantly decrease in KO chimeric mice compared to C chimeric mice 7 days after cSVD (C vs MDP, M_rank_ = 4.914, P = 0.3478; C vs KO, M_rank_ =8.548, P = 0.012) (Figure 4(c)). However, density of IBA1^+^ cell expressing CD45 (activated microglia/MDMs) remained unchanged among all groups at day 7 (P = 0.6587) (Figure 4(d)). Next, infiltration of non-classical CX3CR1^GFP/+^ monocytes into the ipsilateral hippocampus of chimeric mice was analyzed (Figure 4(e)). As expected, non-classical monocytes were absent in KO chimeric mice, but unexpectedly low infiltration rate was observed in MDP chimeric mice compared to C chimeric mice 7 days after cSVD (C vs MDP, M_rank_ = 8, P = 0.0169; C vs KO, M_rank_ = 10.43, P = 0.0016) (Figure 4(f)). MDP-mediated low infiltration at day 7 was suspected to rather reflect an early infiltration, therefore non-classical monocyte recruitment was assessed 3 days after cSVD in CX3CR1^GFP/+^ mice expressing GFP in microglia and non-classical monocytes. TMEM119 expression was used to discriminate resident microglia (TMEM119^+^/GFP^+^ cells; Yellow) from infiltrating CX3CR1^GFP/+^ monocytes (TMEM119^−^/GFP^+^ cells; Green). Density of TMEM119^−^/CX3CR1^GFP/+^ monocytes in MDP mice was higher compared to C mice (CI [398.8, 1341], P = 0.0051) (Figure 4(g) and (h)). This suggests that MDP promoted a rapid infiltration of non-classical monocytes into the ipsilateral hippocampus. We next analyzed resident microglial reactivity at day 7 in the ipsilateral hippocampus by immunolabeling TMEM119 (Figure 4(i)). Density of TMEM119^+^ microglia in the ipsilateral hippocampus remained similar in all groups (P = 0.1951) (Figure 4(j)), suggesting that CX3CR1 manipulation in monocytes did not affect resident microglia reactivity. Analysis of GFAP immunolabeling (Figure 4(k)), showed similar astrocytic reactivity in all groups (P = 0.7994) (Figure 4(l)). Our results suggest that CX3CR1 manipulation in monocytes modulates their recruitment to the lesion site with limited effects on resident glial cell responses.

### Non-classical monocytes preserve microvascular integrity and stability in cSVD via CX3CR1

**Figure 5.**
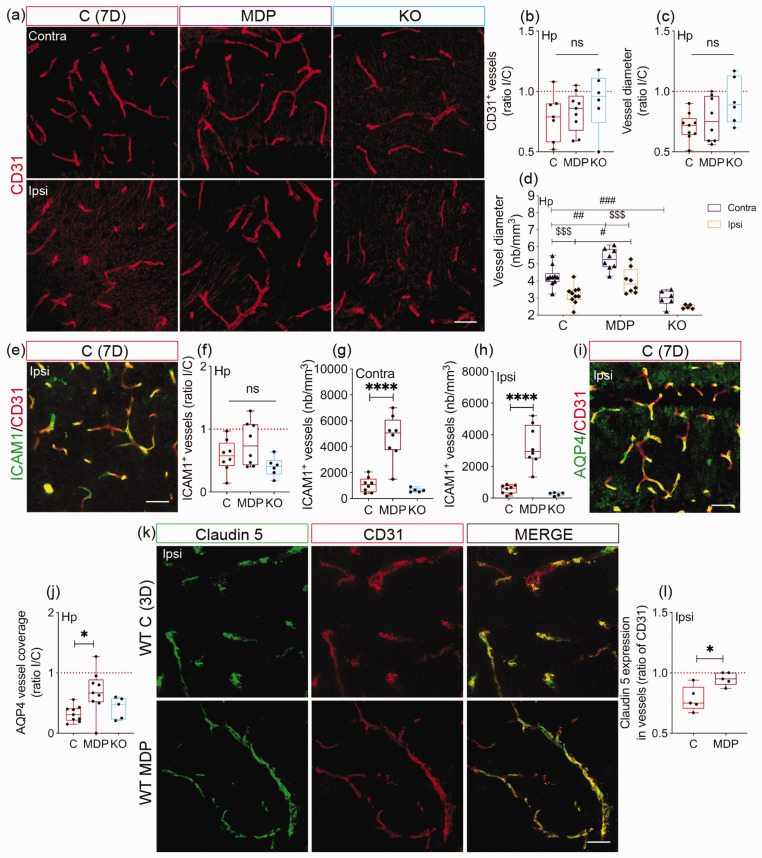
CX3CR1 expression in monocytes is essential for maintaining vascular stability after cSVD. (a) Representative fluorescent images of CD31 immunolabeling in the contralateral and ipsilateral hippocampus of C, MDP and KO chimeric mice 7 days after cSVD. (b) Analysis of CD31^+^ vessel density in the hippocampus 7 days after cSVD, shown as ipsilateral/contralateral (I/C) ratio. (c) Analysis of CD31^+^ vessel average diameter 7 days post-cSVD, shown as I/C ratio. (d) Analysis of the effects of experimental conditions (C, MDP, MO) and hemispheres (contralateral, ipsilateral) on the average diameter of CD31^+^ vessels. No interaction was reported between the experimental conditions and hemiIeres. (e) Representative fluorescent image of ICAM1 immunolabeling in CD31^+^ cells in the ipsilateral hippocampus of C chimeric mice 7 days after cSVD. (f) Analysis of ICAM1 expression in CD31^+^ cells in the hippocampus 7 days after cSVD, shown as I/C ratio. Analysis of ICAM1 absolute density in (g) contralateral and (h) ipsilateral hippocampus 7 days after cSVD. (i) Representative fluorescent image of AQP4 colocalization with CD31^+^ vessels in the ipsilateral hippocampus 7 days after cSVD. (j) Analysis of AQP4 coverage of the CD31^+^ vessels, shown as I/C ratio. (k) Representative fluorescent images of claudin 5 and CD31 co-immunolabeling in the ipsilateral hippocampus of WT C and MDP mice 3 days after cSVD. (l) Analysis of claudin 5 expression (density) in CD31^+^ vessels, shown as ratio of CD31 structures. Data are boxplot with min/max and mean±SD (d) (n = 3–9 animals/group). I/C ratio = 1 indicates similar pattern in ipsilateral and contralateral hemispheres. *P < 0.05/****P < 0.0001 compared to C chimeric mice or WT C mice (unpaired two-tailed *t*-test or one-way ANOVA); ^#^P < 0.05/^##^P < 0.01/^###^P < 0.001 comparison among the experimental groups (C, MDP, KO (row factor)) independently upon hemispheric injury and ^$$$^P < 0.001 comparisons between the hemispheres (column factor) independently upon the experimental conditions (two-way ANOVA (d)). Statistical summary is provided in (Supplementary Material 2). Scale bar = 50 µm. D, days.

Since non-classical monocytes maintain vascular homeostasis,^11,18,19^ we aimed to evaluate whether modulating their function through manipulation of CX3CR1 expression affects the microvasculature in cSVD. Density and diameter of CD31^+^ vessels were assessed in the hippocampus of chimeric mice 7 days after cSVD (Figure 5(a)). Density of CD31^+^ vessels remained unchanged in the ipsilateral hippocampus of all groups (P = 0.6104) (Figure 5(b)). The average diameter of CD31^+^ vessels tended to be less reduced in the ipsilateral hippocampus of KO chimeric mice (P = 0.0618; C = 0.7078 ± 0.1132; MDP = 0.7688 ± 0.1857; KO = 0.9217 ± 0.1893) (Figure 5 (c)). There was no interaction between the experimental conditions (C, MDP, MO) and hemispheres (contralateral, ipsilateral) on CD31^+^ vessel average diameter (two-way ANOVA, P = 0.2341). Nonetheless, there were independent effects among the experimental conditions (row factor, P < 0.0001) and between the hemispheres (column factor, P < 0.0001) (Figure 5(d)). The average diameter of CD31^+^ vessels increased in both contralateral and ipsilateral hippocampus of MDP chimeric mice, while it was reduced in the contralateral hippocampus of KO chimeric mice compared to C chimeric mice (Contra: C vs MDP, CI [−1.683, −0.3413], P = 0.002; C vs KO, CI [0.5428, 2.003], P = 0.0004/Ipsi: C vs MDP, CI [−1.466, −0.1243], P = 0.0169). This suggests that the basal vascular response at the hippocampus is compromised in KO chimeric mice independently of cSVD-mediated hemispheric injury. Furthermore, the average CD31^+^ vessel diameter was decreased in C and MDP chimeric mice but not in KO chimeric animals (C, CI [0.4257, 1.72], P = 0.0005; MDP, CI [0.5663, 2.014], P = 0.0002; KO, CI [−0.3675, 1.385], P = 0.3986) (Figure 5(d)). Next, vascular expression of ICAM1, which regulates monocyte trafficking, was assessed (Figure 5(e)).^14,44^ Ratio of ICAM1^+^ vessel density in the ipsilateral compared to the contralateral hippocampus decreased in C and KO chimeric mice, but remained similar in MDP chimeric mice (P = 0.0644, C = 0.5763 ± 0.2578; MDP = 0.7725 ± 0.35; KO = 0.4 ± 0.1543) (Figure 5(f)). Absolute density of ICAM1^+^ vessels increased in both contralateral (C vs MDP, CI [−5107, −2216], P < 0.0001) (Figure 5(g)) and ipsilateral hippocampus (C vs MDP, CI [−3820, −1694], P < 0.0001) (Figure 5(h)) of MDP chimeric mice compared to C chimeric mice 7 days after cSVD, outlining an overall increased ICAM1 expression upon MDP administration. Microvascular stability depends upon adequate coverage with astrocyte endfeet.^45^ AQP4^+^ astrocyte endfeet coverage of CD31^+^ vessels (Figure 5(i)) decreased in the ipsilateral hippocampus of C and KO chimeric mice, but was rescued in MDP chimeric mice (C vs MDP, CI [−0.6626, −0.06626], P = 0.0152; C vs KO, CI [−0.4526, 0.2530], P = 0.7573) (Figure 5(j)). The tight junction protein claudin 5 maintains cerebrovascular tightness.^46,47^ Claudin 5 expression in CD31^+^ vessels (Figure 5(k)) was rescued in the ipsilateral hippocampus of WT MDP mice compared to WT C mice (CI [0.04382, 0.2842], P = 0.0137) (Figure 5(l)). These results indicate that CX3CR1^GFP/+^ monocytes preserve microvascular structural integrity in cSVD.

### Stimulation of non-classical monocytes improves brain perfusion after cSVD associated with secretion of pro-angiogenic factors

**Figure 6.**
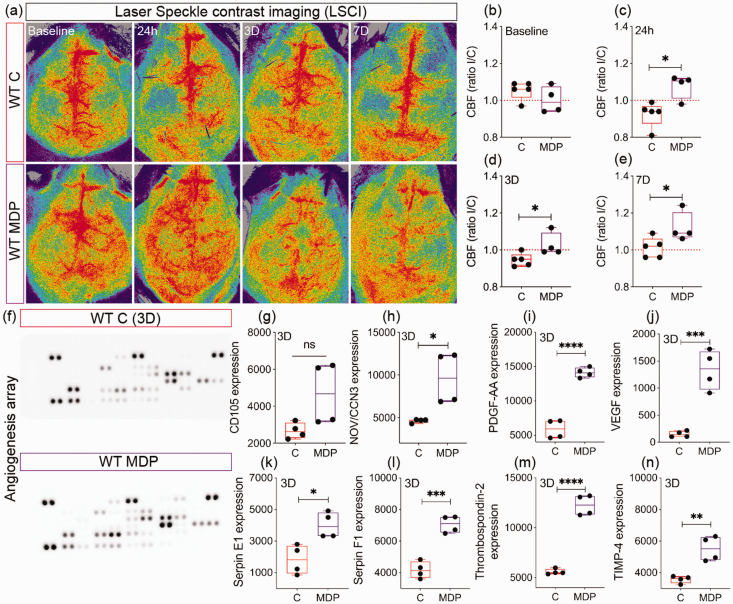
Non-classical monocytes promote vascular function in cSVD. (a) Representative LSCI showing the temporal changes in the CBF of WT C and MDP mice up to 7 days post-cSVD. Analysis of CBF (b) at baseline (c) 24 h, (d) 3 days and (e) 7 days post-cSVD, shown as ipsilateral/contralateral (I/C) ratio. (f) Representative images of the Proteome Profiler mouse angiogenesis array membranes profiling expression of proteins implicated in vascular remodeling in the serum of CX3CR1^GFP/+^ C and MDP mice 3 days post-cSVD. Analysis of the expression (optical density) of (g) CD105, (h) NOV/CCN3, (i) PDGF-AA and (j) VEGF, (k) serpin E1, (l) serpin F1, (m) thrombospondin 2 and (n) TIMP-4 in the serum of CX3CR1^GFP/+^ C and MDP mice. Data are boxplot with min/max (n = 4–5 animals/group; n = 4 dots/experimental condition). I/C ratio = 1 indicates similar pattern in ipsilateral and contralateral hemispheres. *P < 0.05/**P < 0.01/***P < 0.001/^****^P < 0.0001 compared to WT C mice or CX3CR1^GFP/+^ C mice (unpaired two-tailed *t*-test). Statistical summary is provided in (Supplementary Material 2). D, days.

Since our results showed that non-classical monocytes preserved microvascular architecture, we aimed to evaluate the functional consequences on CBF. This was achieved using LSCI in WT C and MDP mice (Figure 6(a)). CBF values were identical in both hemispheres in both groups at baseline (CI [−0.1459, 0.04291], P = 0.2381) (Figure 6(b)). Nonetheless, CBF values slightly decreased 24 h after cSVD in the ipsilateral hemisphere of WT C mice, indicative of a mild hypoperfusion, whereas it increased in WT MDP mice (CI [0.04527, 0.2577], P = 0.0119) (Figure 6(c)), outlining an enhanced perfusion. CBF values in the ipsilateral hemisphere remained slightly decreased at day 3 in WT C mice, whereas it remained elevated in WT MDP mice (CI [0.004266, 0.1587], P = 0.0413) (Figure 6(d)). Interestingly, WT C and MDP mice showed identical CBF values in both hemispheres at day 7, whereas CBF values improved in the ipsilateral hemisphere in WT MDP mice (CI [0.001288, 0.2147], P = 0.0479) (Figure 6(e)). Finally, expression of various factors involved in vascular remodeling was assessed in the blood circulation of CX3CR1^GFP/+^ C and MDP mice 3 days after cSVD (Figure 6(f)), the time point at which MDP-mediated rapid non-classical CX3CR1^+^ monocytes infiltration was observed. MDP increased the levels of pro-angiogenic factors, namely CD105 (CI [−131.7, 4139], P = 0.0614; C = 2678 ± 432; MDP = 4682 ± 1691) (Figure 6(g)), nephroblastoma overexpressed/cellular communication network factor-3 (NOV/CCN3) (CI [1292, 8710], P = 0.0164) (Figure 6(h)), platelet-derived growth factor (PDGF)-AA (CI [6359, 10130], P < 0.0001) (Figure 6(i)), and vascular endothelial growth factor (VEGF) (CI [731.8, 1641], P = 0.0007) (Figure 6(j)). Similarly, MDP increased the expression of matrix stabilizer factors, namely serpin E1 (CI [683.6, 3704], P = 0.012) (Figure 6(k)), serpin F1 (CI [1962, 3802], P = 0.0003) (Figure 6(l)), thrombospondin-2 (CI [5371, 7972], P < 0.0001) (Figure 6(m)) and tissue inhibitor of metalloproteinase (TIMP)-4 (CI [953.8, 2943], P = 0.003) (Figure 6(n)). Our results indicate that CX3CR1^GFP/+^ monocyte stimulation after cSVD attenuates CBF deregulation associated with the release of vascular protective factors in the blood circulation.

## Discussion

Monocytes are implicated in tissue repair in various pathologies.^11,48^ Our study aimed to investigate role of non-classical CX3CR1 monocytes in cSVD pathobiology and therapy, with an emphasis on hippocampus, a structure critically involved in memory and anxiety.^6,41,49^ Herein, we performed a longitudinal analysis of structural damage and infiltration of CX3CR1^GFP/+^ monocytes 3 and 7 days as well as 1 month after cSVD. We found that hippocampal microinfarcts are prevalent in cSVD, and associated with neuronal degeneration and IgG extravasation, peaking at day 3, and gradually returning to baseline 1 month later. This suggests a transient neurodegenerative response and BBB disruption, which is in line with recent reports.^6,7^ Upon brain injury, microglia get activated and contribute to the repair process via elimination of cell debris and secretion of trophic factors.^42,43^ IBA1^+^ cell reactivity peaked at day 7, and gradually decreased at 1 month. However, an important proportion of CD45^+^ cells did not express IBA1 between days 3 and 7, outlining infiltration of myeloid cells. Furthermore, mRNA levels of TREM2 that regulates monocyte recruitment, and of IGF1 that promotes microvascular stability, were elevated at the lesion site, coinciding with CX3CR1^GFP/+^ monocytes massive recruited. Monocytes are the most abundant cells that infiltrate the lesion site between days 3 and 7 upon ischemic insults.^50^ Yet, role of CX3CR1^+^ non-classical monocytes in cSVD remains elusive. Using chimeric mice, we showed CX3CR1^GFP/+^ monocyte infiltrate the lesion site 3 days after cSVD, peaking at day 7, and vanishing at 1 month. These observations are in line with previous reports outlining a transient infiltration of monocytes into ischemic tissue.^6,51–53^ CX3CR1^GFP/+^ monocyte infiltration coincided with the attenuation of neuronal degeneration and BBB disruption, outlining their possible contribution to the repair process. The transient infiltration suggests that non-classical monocytes acted via mechanisms that do not involve colonization of the injured brain.

Our group has recently reported that monocyte subsets are differentially regulated after cSVD.^6^ To characterize the role of non-classical monocytes, we generated chimeric mice in which CX3CR1 expression in monocytes was manipulated. Frequencies of different monocyte subsets remained unchanged at baseline, indicating that CX3CR1 depletion does not affect monocyte physiological distribution.^6,54^ Ly6C^high^ CX3CR1^−^ monocyte frequency increased in KO chimeric mice 3 days upon cSVD, whereas that of Ly6C^low^CX3CR1^+^ monocytes decreased. This outlines an impaired Ly6C^low^CX3CR1^+^ monocyte generation in KO chimeric mice from Ly6C^high^ CX3CR1^−^ monocytes. Ly6C^low^CX3CR1^+^ monocyte frequency increased in MDP chimeric mice 7 days after cSVD, whereas Ly6C^high^CX3CR1^−^ subset frequency decreased, highlighting an efficient MDP-mediated conversion of Ly6C^high^ monocytes into Ly6C^low^ subset.^48,55,56^ Frequency of Ly6C^low^CX3CR1^+^ monocytes in KO chimeric mice remained unchanged at day 7, possibly translating an impaired cell response. Indeed, CX3CR1 signaling deregulation alters monocyte trafficking and recruitment into the injured tissue.^48,57^ Neutrophil frequency in KO chimeric mice after cSVD was increased, which could be associated with CX3CR1 dysfunction in monocytes that regulate neutrophil trafficking.^58^

Dysfunctional non-classical monocytes in KO chimeric mice exacerbated neuronal loss and aggravated cognitive deficits after cSVD. These observations are in line with previous reports indicating that CX3CR1 depletion in monocytes exacerbates neuronal loss during excitotoxicity.^20^ Nonetheless, stimulation of CX3CR1^GFP/+^ monocyte generation using MDP promoted neuronal survival, reduced vascular permeability and improved cognitive functions after cSVD. These findings unravel a previously undescribed role of non-classical monocytes in rescuing salvageable neurons and attenuating cognitive deficits after cSVD. It is in line with recent reports showing that stimulation of non-classical monocytes reduces neuronal loss after cerebral ischemia^36^ and decelerates Alzheimer’s disease (AD)-like pathology progression.^34^

Ischemia activates resident microglia and triggers the recruitment of monocytes that differentiate into MDMs.^6,42,43,59^ Here, we showed that activation of resident TMEM119^+^ microglia was not affected by CX3CR1 manipulation in monocytes. As expected, GFP^+^ cells were absent in the ipsilateral hippocampus of KO chimeric mice. Unexpectedly, CX3CR1^GFP/+^ monocyte presence at the lesion site was reduced in MDP chimeric mice.^48,57^ Using CX3CR1^GFP/+^ mice in which resident microglia and circulating non-classical monocytes express GFP, we reported early infiltration of non-resident TMEM119^−^ CX3CR1^+^ monocytes at the lesion site 3 days after cSVD upon MDP administration.

Salvage of neuronal survival depends upon microvascular integrity.^60,61^ Ischemia-mediated vascular deregulation exacerbates neuronal death, whereas restoration of vascular function promotes survival.^62^ cSVD reduced vascular density in the ipsilateral hippocampus, which was exacerbated in KO chimeric mice. Furthermore, average vessel diameter was reduced in the ipsilateral hippocampus but preserved in MDP chimeric mice. Microvascular rarefaction plays a key role in dementia etiology.^63^ Our observations suggest that non-classical monocytes prevent microvascular rarefaction upon cSVD, which is in line with their role in vascular maintenance.^63,64^ MDP increased ICAM1 expression in endothelial cells. As ICAM1 regulates leukocyte recruitment to the inflammation site,^65^ it could account for MDP-mediated early CX3CR1^GFP/+^ monocyte infiltration after cSVD. ICAM1 might facilitate monocyte infiltration to partake in tissue remodeling. Indeed, neutralization of ICAM1 after stroke could worsen ischemic damage by possibly impairing the contribution of CX3CR1^+^ monocytes to the repair process.^66,67^ Astrocyte endfeet optimal interaction with endothelial cells is required to maintain microvascular integrity.^68^ Astrocytic AQP4 fine-tunes brain homeostasis, hippocampus-dependent learning and memory processes, and helps resolving microinfarcts.^67,69–71^ Claudin 5 maintains brain vascular integrity.^46,47^ MDP improved endothelial coverage with AQP4^+^ astrocyte endfeet in the ipsilateral hippocampus after cSVD, as well as claudin 5 expression in vessels. Thus, our results suggest that CX3CR1^GFP/+^ monocyte-mediated microvascular preservation is associated with enhanced astrocyte endfeet coverage and tight junction promotion. Importantly, the improved structural integrity of the vasculature was associated with a preserved CBF in the ipsilateral hemisphere at day 7 after cSVD. These changes were accompanied by the release of pro-angiogenic factors in the blood circulation upon MDP administration, among which are, CD105, a component of endothelial nitric oxide synthase (eNOS) pathway,^72^ NOV/CCN3, an angiogenesis inducer,^73^ as well as PDGF-AA/VEGF, potent angiogenic factors.^61,74^ Furthermore, MDP increased the release of factors involved in matrix stabilization, namely serpin E1, an extracellular matrix proteolysis fine-tuner,^55^ serpin F1, angiogenic vessel stabilizer,^56^ as well as thrombospondin 2 and TIMP4, regulators of matrix metalloproteinases (MMPs) activity.^75,76^ Although MDP is a NOD2 modulator in immune cells, its direct effects on endothelial cells cannot be excluded.

**Figure 7.**
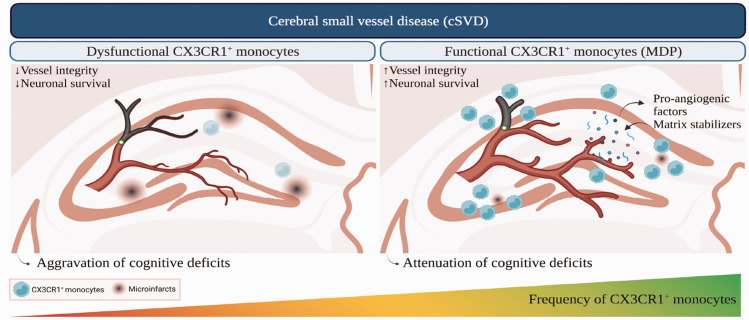
A scheme illustrating the role of non-classical monocytes in the pathobiology of cSVD. cSVD associated with the sporadic micro-occlusion of cerebral arterioles generates microinfarcts in different brain regions, including the hippocampus, leading to cognitive decline. CX3CR1^GFP/+^ monocytes are recruited to the lesion site to partake in neurovascular repair after cSVD. Alteration of CX3CR1 function in monocytes, exemplified by CX3CR1 depletion in circulating monocytes in CX3CR1^GFP/GFP^ mice, exacerbates neuronal damage and aggravates cognitive deficits by impairing vascular integrity. Stimulation of CX3CR1^GFP/+^ monocyte generation via the systemic administration of MDP enhances neuronal survival and attenuates cognitive deficits by preserving vascular structure and function, translated by enhanced brain perfusion, and promoting the systemic release of pro-angiogenic molecules and matrix stabilizers involved in fine-tuning vascular remodeling. Created with BioRender.com.

Herein, we provide new insights into the role of CX3CR1^GFP/+^ monocytes in preserving neurovascular functions and attenuating cognitive decline upon cSVD (Figure 7). These results suggest that strategies aiming to stimulate non-classical monocyte generation constitute a promising approach to promote neurovascular maintenance and cognitive function in cSVD.

## Supplemental Material

sj-pdf-1-jcb-10.1177_0271678X231183742 - Supplemental material for Non-classical monocytes promote neurovascular repair in cerebral small vessel disease associated with microinfarctions via CX3CR1 Click here for additional data file. Supplemental material, sj-pdf-1-jcb-10.1177_0271678X231183742 for Non-classical monocytes promote neurovascular repair in cerebral small vessel disease associated with microinfarctions via CX3CR1 by Sarah Lecordier, Romain Menet, Anne-Sophie Allain and Ayman ElAli in Journal of Cerebral Blood Flow & Metabolism

sj-xlsx-2-jcb-10.1177_0271678X231183742 - Supplemental material for Non-classical monocytes promote neurovascular repair in cerebral small vessel disease associated with microinfarctions via CX3CR1 Click here for additional data file. Supplemental material, sj-xlsx-2-jcb-10.1177_0271678X231183742 for Non-classical monocytes promote neurovascular repair in cerebral small vessel disease associated with microinfarctions via CX3CR1 by Sarah Lecordier, Romain Menet, Anne-Sophie Allain and Ayman ElAli in Journal of Cerebral Blood Flow & Metabolism

## References

[1] Wardlaw JM Smith C Dichgans M. Mechanisms of sporadic cerebral small vessel disease: insights from neuroimaging. Lancet Neurol 2013; 12: 483–497. 23602162 10.1016/S1474-4422(13)70060-7 PMC3836247

[2] Wardlaw JM Smith E Biessels GJ , et al. Neuroimaging standards for research into small vessel disease and its contribution to ageing and neurodegeneration. Lancet Neurol 2013; 12: 822–838. 23867200 10.1016/S1474-4422(13)70124-8 PMC3714437

[3] Lecordier S Manrique-Castano D El Moghrabi Y , et al. Neurovascular alterations in vascular dementia: emphasis on risk factors. Front Aging Neurosci 2021; 13: 727590. 34566627 10.3389/fnagi.2021.727590 PMC8461067

[4] Pantoni L. Cerebral small vessel disease: from pathogenesis and clinical characteristics to therapeutic challenges. Lancet Neurol 2010; 9: 689–701. 20610345 10.1016/S1474-4422(10)70104-6

[5] Li Q Yang Y Reis C , et al. Cerebral small vessel disease. Cell Transplant 2018; 27: 1711–1722. 30251566 10.1177/0963689718795148 PMC6300773

[6] Lecordier S Pons V Rivest S , et al. Multifocal cerebral microinfarcts modulate early Alzheimer’s disease pathology in a sex-dependent manner. Front Immunol 2021; 12: 813536. 35173711 10.3389/fimmu.2021.813536 PMC8841345

[7] Silasi G She J Boyd JD , et al. A mouse model of small-vessel disease that produces brain-wide-identified microocclusions and regionally selective neuronal injury. J Cereb Blood Flow Metab 2015; 35: 734–738. 25690472 10.1038/jcbfm.2015.8 PMC4420872

[8] Balbi M Vanni MP Vega MJ , et al. Longitudinal monitoring of mesoscopic cortical activity in a mouse model of microinfarcts reveals dissociations with behavioral and motor function. J Cereb Blood Flow Metab 2019; 39: 1486–1500. 29521138 10.1177/0271678X18763428 PMC6681536

[9] Iadecola C. The neurovascular unit coming of age: a journey through neurovascular coupling in health and disease. Neuron 2017; 96: 17–42. 28957666 10.1016/j.neuron.2017.07.030 PMC5657612

[10] Zlokovic BV. The blood-brain barrier in health and chronic neurodegenerative disorders. Neuron 2008; 57: 178–201. 18215617 10.1016/j.neuron.2008.01.003

[11] Michaud J-P Bellavance M-A Préfontaine P , et al. Real-time in vivo imaging reveals the ability of monocytes to clear vascular amyloid beta. Cell Rep 2013; 5: 646–653. 24210819 10.1016/j.celrep.2013.10.010

[12] Vlacil A-K Schuett J Schieffer B , et al. Variety matters: diverse functions of monocyte subtypes in vascular inflammation and atherogenesis. Vascul Pharmacol 2019; 113: 9–19. 30553027 10.1016/j.vph.2018.12.002

[13] Pons V Rivest S. Targeting systemic innate immune cells as a therapeutic avenue for Alzheimer disease. Pharmacol Rev 2022; 74: 1–17. 34987086 10.1124/pharmrev.121.000400

[14] Min J-K Kim Y-M Kim SW , et al. TNF-related activation-induced cytokine enhances leukocyte adhesiveness: induction of ICAM-1 and VCAM-1 via TNF receptor-associated factor and protein kinase C-dependent NF-kappaB activation in endothelial cells. J Immunol 2005; 175: 531–540. 15972689 10.4049/jimmunol.175.1.531

[15] ElAli A Jean LeBlanc N. The role of monocytes in ischemic stroke pathobiology: new avenues to explore. Front Aging Neurosci 2016; 8: 29. 26941641 10.3389/fnagi.2016.00029 PMC4761876

[16] Auffray C Sieweke MH Geissmann F. Blood monocytes: development, heterogeneity, and relationship with dendritic cells. Annu Rev Immunol 2009; 27: 669–692. 19132917 10.1146/annurev.immunol.021908.132557

[17] Crane MJ Daley JM van Houtte O , et al. The monocyte to macrophage transition in the murine sterile wound. PLoS One 2014; 9: e86660. 24466192 10.1371/journal.pone.0086660 PMC3899284

[18] Carlin LM Stamatiades EG Auffray C , et al. Nr4a1-dependent Ly6C(low) monocytes monitor endothelial cells and orchestrate their disposal. Cell 2013; 153: 362–375. 23582326 10.1016/j.cell.2013.03.010 PMC3898614

[19] Thériault P ElAli A Rivest S. The dynamics of monocytes and microglia in Alzheimer’s disease. Alzheimers Res Ther 2015; 7: 41. 25878730 10.1186/s13195-015-0125-2 PMC4397873

[20] Bellavance M-A Gosselin D Yong VW , et al. Patrolling monocytes play a critical role in CX3CR1-mediated neuroprotection during excitotoxicity. Brain Struct Funct 2015; 220: 1759–1776. 24706067 10.1007/s00429-014-0759-z

[21] Kumar AHS Martin K Turner EC , et al. Role of CX3CR1 receptor in monocyte/macrophage driven neovascularization. PLoS One 2013; 8: e57230. 23437346 10.1371/journal.pone.0057230 PMC3578809

[22] Olingy CE San Emeterio CL Ogle ME , et al. Non-classical monocytes are biased progenitors of wound healing macrophages during soft tissue injury. Sci Rep 2017; 7: 447. 28348370 10.1038/s41598-017-00477-1 PMC5428475

[23] Wolf AA Yáñez A Barman PK , et al. The ontogeny of monocyte subsets. Front Immunol 2019; 10: 1642. 31379841 10.3389/fimmu.2019.01642 PMC6650567

[24] Geissmann F Jung S Littman DR. Blood monocytes consist of two principal subsets with distinct migratory properties. Immunity 2003; 19: 71–82. 12871640 10.1016/s1074-7613(03)00174-2

[25] Tacke F Alvarez D Kaplan TJ , et al. Monocyte subsets differentially employ CCR2, CCR5, and CX3CR1 to accumulate within atherosclerotic plaques. J Clin Invest 2007; 117: 185–194. 17200718 10.1172/JCI28549 PMC1716202

[26] Li L Huang L Sung S-SJ , et al. The chemokine receptors CCR2 and CX3CR1 mediate monocyte/macrophage trafficking in kidney ischemia-reperfusion injury. Kidney Int 2008; 74: 1526–1537. 18843253 10.1038/ki.2008.500 PMC2652647

[27] Landsman L Bar-On L Zernecke A , et al. CX3CR1 is required for monocyte homeostasis and atherogenesis by promoting cell survival. Blood 2009; 113: 963–972. 18971423 10.1182/blood-2008-07-170787

[28] Jay TR Miller CM Cheng PJ , et al. TREM2 deficiency eliminates TREM2+ inflammatory macrophages and ameliorates pathology in Alzheimer’s disease mouse models. J Exp Med 2015; 212: 287–295. 25732305 10.1084/jem.20142322 PMC4354365

[29] Hayes CA Ashmore BG Vijayasankar A , et al. Insulin-like growth factor-1 differentially modulates glutamate-induced toxicity and stress in cells of the neurogliovascular unit. Front Aging Neurosci 2021; 13: 751304. 34887742 10.3389/fnagi.2021.751304 PMC8650493

[30] O'Donnell SL Frederick TJ Krady JK , et al. IGF-I and microglia/macrophage proliferation in the ischemic mouse brain. Glia 2002; 39: 85–97. 12112378 10.1002/glia.10081

[31] Piec P-A Pons V Rivest S. Triggering innate immune receptors as new therapies in Alzheimer’s disease and multiple sclerosis. Cells 2021; 10: 2164. 34440933 10.3390/cells10082164 PMC8393987

[32] Lessard A-J LeBel M Egarnes B , et al. Triggering of NOD2 receptor converts inflammatory Ly6Chigh into Ly6Clow monocytes with patrolling properties. Cell Rep 2017; 20: 1830–1843. 28834747 10.1016/j.celrep.2017.08.009

[33] Hanna RN Carlin LM Hubbeling HG , et al. The transcription factor NR4A1 (Nur77) controls bone marrow differentiation and the survival of Ly6C- monocytes. Nat Immunol 2011; 12: 778–785. 21725321 10.1038/ni.2063 PMC3324395

[34] Al-Onaizi MA Thériault P Lecordier S , et al. Early monocyte modulation by the non-erythropoietic peptide ARA 290 decelerates AD-like pathology progression. Brain Behav Immun 2022; 99: 363–382. 34343617 10.1016/j.bbi.2021.07.016

[35] Pons V Laflamme N Préfontaine P , et al. Role of macrophage colony-stimulating factor receptor on the proliferation and survival of microglia following systemic nerve and cuprizone-induced injuries. Front Immunol 2020; 11: 47. 32082318 10.3389/fimmu.2020.00047 PMC7001158

[36] Thériault P Le Béhot A ElAli A , et al. Sub-acute systemic erythropoietin administration reduces ischemic brain injury in an age-dependent manner. Oncotarget 2016; 7: 35552–35561. 27248662 10.18632/oncotarget.9652 PMC5094944

[37] Pons V Lévesque P Plante M-M , et al. Conditional genetic deletion of CSF1 receptor in microglia ameliorates the physiopathology of Alzheimer’s disease. Alzheimers Res Ther 2021; 13: 8. 33402196 10.1186/s13195-020-00747-7 PMC7783991

[38] Menet R Bourassa P Calon F , et al. Dickkopf-related protein-1 inhibition attenuates amyloid-beta pathology associated to Alzheimer’s disease. Neurochem Int 2020; 141: 104881. 33068684 10.1016/j.neuint.2020.104881

[39] Walf AA Frye CA. The use of the elevated plus maze as an assay of anxiety-related behavior in rodents. Nat Protoc 2007; 2: 322–328. 17406592 10.1038/nprot.2007.44 PMC3623971

[40] Jean LeBlanc N Menet R Picard K , et al. Canonical wnt pathway maintains blood-brain barrier integrity upon ischemic stroke and its activation ameliorates tissue plasminogen activator therapy. Mol Neurobiol 2019; 56: 6521–6538. 30852795 10.1007/s12035-019-1539-9

[41] Bannerman DM Rawlins JNP McHugh SB , et al. Regional dissociations within the hippocampus–memory and anxiety. Neurosci Biobehav Rev 2004; 28: 273–283. 15225971 10.1016/j.neubiorev.2004.03.004

[42] Neumann H Kotter MR Franklin RJM. Debris clearance by microglia: an essential link between degeneration and regeneration. Brain 2009; 132: 288–295. 18567623 10.1093/brain/awn109 PMC2640215

[43] Werner Y Mass E Ashok Kumar P , et al. Cxcr4 distinguishes HSC-derived monocytes from microglia and reveals monocyte immune responses to experimental stroke. Nat Neurosci 2020; 23: 351–362. 32042176 10.1038/s41593-020-0585-y PMC7523735

[44] Gerhardt T Ley K. Monocyte trafficking across the vessel wall. Cardiovasc Res 2015; 107: 321–330. 25990461 10.1093/cvr/cvv147 PMC4592323

[45] Mathiisen TM Lehre KP Danbolt NC , et al. The perivascular astroglial sheath provides a complete covering of the brain microvessels: an electron microscopic 3D reconstruction. Glia 2010; 58: 1094–1103. 20468051 10.1002/glia.20990

[46] Lv J Hu W Yang Z , et al. Focusing on claudin-5: a promising candidate in the regulation of BBB to treat ischemic stroke. Prog Neurobiol 2018; 161: 79–96. 29217457 10.1016/j.pneurobio.2017.12.001

[47] Paul D Cowan AE Ge S , et al. Novel 3D analysis of claudin-5 reveals significant endothelial heterogeneity among CNS microvessels. Microvasc Res 2013; 86: 1–10. 23261753 10.1016/j.mvr.2012.12.001 PMC3570614

[48] Auffray C Fogg D Garfa M , et al. Monitoring of blood vessels and tissues by a population of monocytes with patrolling behavior. Science 2007; 317: 666–670. 17673663 10.1126/science.1142883

[49] Duncombe J Kitamura A Hase Y , et al. Chronic cerebral hypoperfusion: a key mechanism leading to vascular cognitive impairment and dementia. Closing the translational gap between rodent models and human vascular cognitive impairment and dementia. Clin Sci (Lond) 2017; 131: 2451–2468. 28963120 10.1042/CS20160727

[50] Beuker C Strecker J-K Rawal R , et al. Immune cell infiltration into the brain after ischemic stroke in humans compared to mice and rats: a systematic review and meta-analysis. Transl Stroke Res 2021; 12: 976–990. 33496918 10.1007/s12975-021-00887-4 PMC8557159

[51] Schilling M Strecker JK Schäbitz WR , et al. Effects of monocyte chemoattractant protein 1 on blood-borne cell recruitment after transient focal cerebral ischemia in mice. Neuroscience 2009; 161: 806–812. 19374937 10.1016/j.neuroscience.2009.04.025

[52] Schilling M Besselmann M Müller M , et al. Predominant phagocytic activity of resident microglia over hematogenous macrophages following transient focal cerebral ischemia: an investigation using green fluorescent protein transgenic bone marrow chimeric mice. Exp Neurol 2005; 196: 290–297. 16153641 10.1016/j.expneurol.2005.08.004

[53] Petry KG Boiziau C Dousset V , et al. Magnetic resonance imaging of human brain macrophage infiltration. Neurotherapeutics 2007; 4: 434–442. 17599709 10.1016/j.nurt.2007.05.005 PMC7479730

[54] Naert G Rivest S. A deficiency in CCR2+ monocytes: the hidden side of Alzheimer’s disease. J Mol Cell Biol 2013; 5: 284–293. 23892208 10.1093/jmcb/mjt028

[55] Wu J Strawn TL Luo M , et al. Plasminogen activator inhibitor-1 inhibits angiogenic signaling by uncoupling vascular endothelial growth factor receptor-2-αVβ3 integrin cross talk. Arterioscler Thromb Vasc Biol 2015; 35: 111–120. 25378411 10.1161/ATVBAHA.114.304554 PMC4270947

[56] Filleur S Nelius T de Riese W , et al. Characterization of PEDF: a multi-functional serpin family protein. J Cell Biochem 2009; 106: 769–775. 19180572 10.1002/jcb.22072

[57] Wohleb ES Powell ND Godbout JP , et al. Stress-induced recruitment of bone marrow-derived monocytes to the brain promotes anxiety-like behavior. J Neurosci 2013; 33: 13820–13833. 23966702 10.1523/JNEUROSCI.1671-13.2013 PMC3755721

[58] Kreisel D Nava RG Li W , et al. In vivo two-photon imaging reveals monocyte-dependent neutrophil extravasation during pulmonary inflammation. Proc Natl Acad Sci U S A 2010; 107: 18073–18078. 20923880 10.1073/pnas.1008737107 PMC2964224

[59] Guruswamy R ElAli A. Complex roles of microglial cells in ischemic stroke pathobiology: new insights and future directions. Int J Mol Sci 2017; 18 10.3390/ijms18030496 PMC5372512 28245599

[60] Hermann DM ElAli A. The abluminal endothelial membrane in neurovascular remodeling in health and disease. Sci Signal 2012; 5: re4. 22871611 10.1126/scisignal.2002886

[61] Lange C Storkebaum E de Almodóvar CR , et al. Vascular endothelial growth factor: a neurovascular target in neurological diseases. Nat Rev Neurol 2016; 12: 439–454. 27364743 10.1038/nrneurol.2016.88

[62] Yemisci M Gursoy-Ozdemir Y Vural A , et al. Pericyte contraction induced by oxidative-nitrative stress impairs capillary reflow despite successful opening of an occluded cerebral artery. Nat Med 2009; 15: 1031–1037. 19718040 10.1038/nm.2022

[63] Hermann DM Zechariah A. Implications of vascular endothelial growth factor for postischemic neurovascular remodeling. J Cereb Blood Flow Metab 2009; 29: 1620–1643. 19654590 10.1038/jcbfm.2009.100

[64] Corliss BA Azimi MS Munson JM , et al. Macrophages: an inflammatory link between angiogenesis and lymphangiogenesis. Microcirculation 2016; 23: 95–121. 26614117 10.1111/micc.12259 PMC4744134

[65] Bui TM Wiesolek HL Sumagin R. ICAM-1: a master regulator of cellular responses in inflammation, injury resolution, and tumorigenesis. J Leukoc Biol 2020; 108: 787–799. 32182390 10.1002/JLB.2MR0220-549R PMC7977775

[66] Wang L Chen Y Feng D , et al. Serum ICAM-1 as a predictor of prognosis in patients with acute ischemic stroke. Biomed Res Int 2021; 2021: 5539304. 33791362 10.1155/2021/5539304 PMC7997739

[67] Jeon H Kim M Park W , et al. Upregulation of AQP4 improves Blood-Brain barrier integrity and perihematomal edema following intracerebral hemorrhage. Neurotherapeutics 2021; 18: 2692–2706. 34545550 10.1007/s13311-021-01126-2 PMC8804112

[68] Abbott NJ Rönnbäck L Hansson E. Astrocyte-endothelial interactions at the blood-brain barrier. Nat Rev Neurosci 2006; 7: 41–53. 16371949 10.1038/nrn1824

[69] Vandebroek A Yasui M. Regulation of AQP4 in the central nervous system. Int J Mol Sci 2020; 21 10.3390/ijms21051603 PMC7084855 32111087

[70] Szu JI Binder DK. The role of astrocytic aquaporin-4 in synaptic plasticity and learning and memory. Front Integr Neurosci 2016; 10: 8. 26941623 10.3389/fnint.2016.00008 PMC4764708

[71] Regenhardt RW Das AS Lo EH , et al. Advances in understanding the pathophysiology of lacunar stroke: a review. JAMA Neurol 2018; 75: 1273–1281. 30167649 10.1001/jamaneurol.2018.1073 PMC7426021

[72] Zhu W Ma L Zhang R , et al. The roles of endoglin gene in cerebrovascular diseases. Neuroimmunol Neuroinflamm 2017; 4: 199–210. 29098173 10.20517/2347-8659.2017.18 PMC5663457

[73] Lin CG Chen C-C Leu S-J , et al. Integrin-dependent functions of the angiogenic inducer NOV (CCN3): implication in wound healing. J Biol Chem 2005; 280: 8229–8237. 15611078 10.1074/jbc.M404903200

[74] Shikada Y Yonemitsu Y Koga T , et al. Platelet-derived growth factor-AA is an essential and autocrine regulator of vascular endothelial growth factor expression in non-small cell lung carcinomas. Cancer Res 2005; 65: 7241–7248. 16103075 10.1158/0008-5472.CAN-04-4171

[75] Fernández CA Moses MA. Modulation of angiogenesis by tissue inhibitor of metalloproteinase-4. Biochem Biophys Res Commun 2006; 345: 523–529. 16682001 10.1016/j.bbrc.2006.04.083

[76] Daniel C Amann K Hohenstein B , et al. Thrombospondin 2 functions as an endogenous regulator of angiogenesis and inflammation in experimental glomerulonephritis in mice. J Am Soc Nephrol 2007; 18: 788–798. 17287428 10.1681/ASN.2006080873

