# Supplementary material for: Non-classical monocytes promote neurovascular repair in cerebral small vessel disease associated with microinfarctions via CX3CR1
Source: J Cereb Blood Flow Metab. 2023 Jun 21;43(11):1873–90. doi: 10.1177/0271678X231183742 (PMC10676133; doi:10.1177/0271678X231183742)
Supplement: sj-pdf-1-jcb-10.1177_0271678X231183742 - Supplemental material for Non-classical monocytes promote neurovascular repair in cerebral small vessel disease associated with microinfarctions via CX3CR1 [file sj-pdf-1-jcb-10.1177_0271678X231183742.pdf]

Non-classical monocytes promote neurovascular repair in cerebral small vessel disease associated with microinfarctions via CX3CR1

Sarah Lecordier, Romain Menet, Anne-Sophie Allain and Ayman ElAli

Supplementary material

Antibody	Species	Dilution	Compagnie	Catalogue #
CD45 APC	Rat	1:100	Biolegend	103112
CD11b Pe Cy7	Rat	1:100	Ebiosciences	25-0112
Ly6C V450	Rat	1:100	BD Bioscience	560594
Ly6G Pe	Rat	1:100	BD bioscience	551461
Live / Dead	Rat	1:100	Life technologies	L23105

Supplementary Table 1. Presentation of the different antibodies used for Flow Cytometry.

Antibody	Species	Dilution	Compagnie	Catalogue #
Primary Antibodies				
NeuN	Chicken	1:1000	EMD Millipore	MAB377
DCX	Rabbit	1:1000	Abcam	ab18723
IBA1	Rabbit	1:1000	WAKO	019-19741
TUJ1	Rabbit	1:500	Abcam	ab18297
MAP2	Mouse	1:500	EMD Millipore	MAB3418
GFAP	Rat	1:500	Invitrogen	13-0300
CD31	Goat	1:250	R&D system	AF3628
CD45	Rat	1:500	BD Biosciences	553076
ICAM1	Goat	1:250	R&D system	AF796
AQP4	Rabbit	1:250	Cell Signalling	59678
CD68	Rat	1:1000	Biorad	MCA1957
TMEM119	Guineapig	1:500	Synaptic system	400 004
Claudin 5	Rabbit	1:250	Abcam	ab15106
Secondary Antibodies				
AffiniPure goat anti-rabbit IgG (H+L)	Goat	1:1000	Invitrogen	A10523
AffiniPure donkey anti-goat IgG (H+L)	Donkey	1:1000	Jackson Immunoresearch	705605003
AffiniPure donkey anti-chicken IgG (H+L)	Donkey	1:1000	EMD Millipore	AP194C
AffiniPure goat anti-rat IgG (H+L)	Goat	1:1000	Invitrogen	A11081
AffiniPure goat anti-mouse IgG (H+L)	Goat	1:1000	Jackson Immunoresearch	111-165-003

Supplementary Table 2. Presentation of the different antibodies used for immunofluorescence.

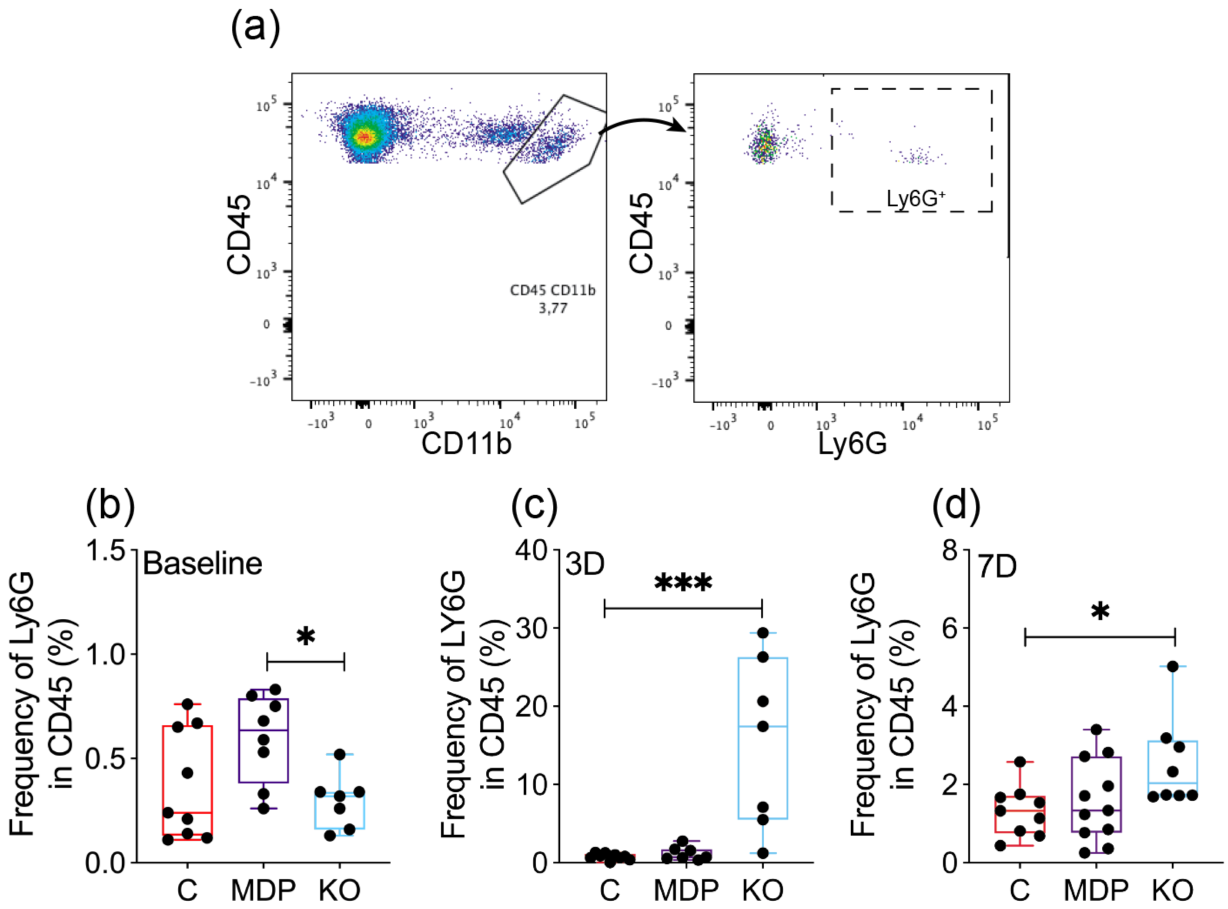

Supplementary Figure 1. The dynamics of neutrophils in the blood circulation is affected by the specific manipulation of CX3CR1 subset and cSVD. (a) Gating strategy used to discriminate neutrophils ($Ly6G^+$) in leukocytes in the blood circulation after cSVD. Flow cytometry analysis of neutrophil frequency at (b) baseline (c) 3 days and (d) 7 days after cSVD. Data are boxplot with min/max (n=7-9 animals/group). $^*P<0.05$/$^{***}P<0.001$ compared to C chimeric mice (one-way ANOVA). Statistical summary is provided in (Supplementary Material 2). D, days.

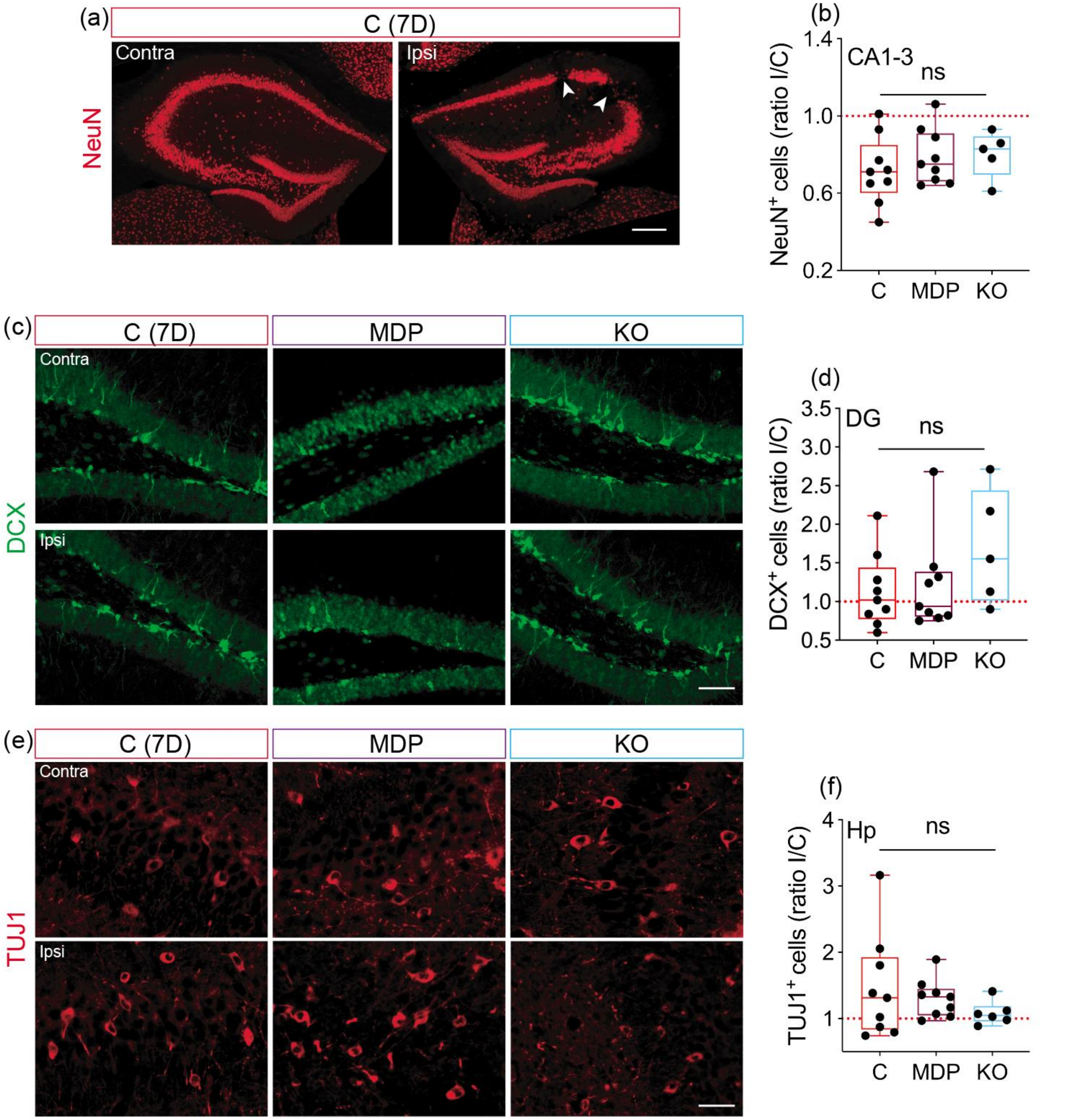

Supplementary Figure 2. Infiltration of non-classical monocytes has limited effects on the neurorestorative responses after cSVD. (a) Representative fluorescent images of NeuN immunolabeling in the contralateral and ipsilateral hippocampus of C chimeric mice 7 days post-cSVD. (b) Stereological analysis of NeuN^{+} cell density in the hippocampus, shown as ipsilateral/contralateral (I/C) ratio. (c) Representative fluorescent images of DCX immunolabeling in the dentate gyrus (DG) of C, MDP and KO

chimeric mice 7 days after cSVD. (d) Stereological analysis of DCX^+ cell density in the DG, shown as I/C ratio. (e) Representative fluorescent images of TUJ1 immunolabeling in the hippocampus of C, MDP and KO chimeric mice 7 days after cSVD. (f) Stereological analysis of $TUJ1^+$ cell density in the hippocampus, shown as I/C ratio. Data are boxplot with min/max (n=5-9 animals/group). I/C ratio = 1 indicates similar pattern in ipsilateral and contralateral hemispheres. Scale bar = 150 µm (a); 200 µm (d, f). Statistical summary is provided in (Supplementary Material 2). D, days.
